# Genomic and transcriptomic analyses reveal distinct biological functions for cold shock proteins (*Vpa*CspA and *Vpa*CspD) in *Vibrio parahaemolyticus* CHN25 during low-temperature survival

**DOI:** 10.1186/s12864-017-3784-5

**Published:** 2017-06-05

**Authors:** Chunhua Zhu, Boyi Sun, Taigang Liu, Huajun Zheng, Wenyi Gu, Wei He, Fengjiao Sun, Yaping Wang, Meicheng Yang, Weicheng Bei, Xu Peng, Qunxin She, Lu Xie, Lanming Chen

**Affiliations:** 1 Key Laboratory of Quality and Safety Risk Assessment for Aquatic Products on Storage and Preservation (Shanghai), China Ministry of Agriculture; College of Food Science and Technology, Shanghai Ocean University, 999 Hu Cheng Huan Road, Shanghai, 201306 People’s Republic of China; 2 College of Information Technology, Shanghai Ocean University, 999 Hu Cheng Huan Road, Shanghai, 201306 People’s Republic of China; 3 Shanghai-MOST Key Laboratory of Disease and Health Genomics, Chinese National Human Genome Centre at Shanghai, Shanghai, 201203 People’s Republic of China; 4 Shanghai Hanyu Bio-lab, 151 Ke Yuan Road, Shanghai, 201203 People’s Republic of China; 5 Shanghai Institute for Food and Drug Control, 1500 Zhang Heng Road, Shanghai, 201203 People’s Republic of China; 6 State Key Laboratory of Agricultural Microbiology, Laboratory of Animal Infectious Diseases, College of Animal Science & Veterinary Medicine, Huazhong Agricultural University, Wuhan, Hubei 430070 People’s Republic of China; 7 Archaea Centre, Department of Biology, University of Copenhagen, Ole Maaløes Vej 5, DK2200, Copenhagen N, Denmark; 8 Shanghai Center for Bioinformation Technology, 1278 Keyuan Road, Shanghai, 201203 People’s Republic of China

**Keywords:** *Vibrio parahaemolyticus*, Complete genome sequence, Cold shock protein, Gene deletion, Transcriptome, Low-temperature adaptation

## Abstract

**Background:**

*Vibrio parahaemolyticus* causes serious seafood-borne gastroenteritis and death in humans. Raw seafood is often subjected to post-harvest processing and low-temperature storage. To date, very little information is available regarding the biological functions of cold shock proteins (CSPs) in the low-temperature survival of the bacterium. In this study, we determined the complete genome sequence of *V. parahaemolyticus* CHN25 (serotype: O5:KUT). The two main CSP-encoding genes (*VpacspA* and *VpacspD*) were deleted from the bacterial genome, and comparative transcriptomic analysis between the mutant and wild-type strains was performed to dissect the possible molecular mechanisms that underlie low-temperature adaptation by *V. parahaemolyticus*.

**Results:**

The 5,443,401-bp *V. parahaemolyticus* CHN25 genome (45.2% G + C) consisted of two circular chromosomes and three plasmids with 4,724 predicted protein-encoding genes. One dual-gene and two single-gene deletion mutants were generated for *VpacspA* and *VpacspD* by homologous recombination. The growth of the Δ*VpacspA* mutant was strongly inhibited at 10 °C, whereas the *VpacspD* gene deletion strongly stimulated bacterial growth at this low temperature compared with the wild-type strain. The complementary phenotypes were observed in the reverse mutants (Δ*VpacspA*-com, and Δ*VpacspD*-com). The transcriptome data revealed that 12.4% of the expressed genes in *V. parahaemolyticus* CHN25 were significantly altered in the Δ*VpacspA* mutant when it was grown at 10 °C. These included genes that were involved in amino acid degradation, secretion systems, sulphur metabolism and glycerophospholipid metabolism along with ATP-binding cassette transporters. However, a low temperature elicited significant expression changes for 10.0% of the genes in the Δ*VpacspD* mutant, including those involved in the phosphotransferase system and in the metabolism of nitrogen and amino acids. The major metabolic pathways that were altered by the dual-gene deletion mutant (Δ*VpacspAD*) radically differed from those that were altered by single-gene mutants. Comparison of the transcriptome profiles further revealed numerous differentially expressed genes that were shared among the three mutants and regulators that were specifically, coordinately or antagonistically modulated by *Vpa*CspA and *Vpa*CspD. Our data also revealed several possible molecular coping strategies for low-temperature adaptation by the bacterium.

**Conclusions:**

This study is the first to describe the complete genome sequence of *V. parahaemolyticus* (serotype: O5:KUT). The gene deletions, complementary insertions, and comparative transcriptomics demonstrate that *Vpa*CspA is a primary CSP in the bacterium, while *Vpa*CspD functions as a growth inhibitor at 10 °C. These results have improved our understanding of the genetic basis for low-temperature survival by the most common seafood-borne pathogen worldwide.

**Electronic supplementary material:**

The online version of this article (doi:10.1186/s12864-017-3784-5) contains supplementary material, which is available to authorized users.

## Background

*Vibrio parahaemolyticus* naturally occurs in marine, estuarine and aquaculture environments worldwide and causes serious seafood-borne gastroenteritis and death in humans, particularly when raw, undercooked or mishandled seafood is consumed [1, 2]. *V. parahaemolyticus* was initially identified in 1950 in Osaka, Japan, where an outbreak of acute gastroenteritis that was caused by the consumption of semidried juvenile sardines sickened 272 people and killed 20 [3]. To date, over eighty *V. parahaemolyticus* serotypes have been described based on the somatic (O) and capsular (K) antigens [1]. Of these serotypes, complete genome sequences have been published for three *V. parahaemolyticus* strains—RIMD2210633 (serotype: O3:K6) [4], BB22OP (serotype: O4:K8) [5] and UCM-V493 (serotype: O2:K28) strains [6]. Additionally, two complete and multiple draft genome sequences for the *V. parahaemolyticus* strains are available in the GenBank database (http://www.ncbi.nlm.nih.gov/genome/) and online (http://www.genomesonline.org) [7–9].

*V. parahaemolyticus* is a gram-negative bacterium that is frequently isolated from raw seafood [2]. Seafood is often subjected to post-harvest processing and low-temperature storage, during which the bacterium is challenged to survive under detrimental cold conditions. Previous studies have indicated that the temperature decrease elicits complex cold shock responses in food-related bacteria (e.g., lactic acid bacteria, food spoilage bacteria and food-borne pathogens), such as the regulation of uptake or synthesis of compatible solutes, DNA supercoiling modifications, membrane fluidity maintenance, and cold shock protein (CSP) production (for a review, see [10]).

CSPs comprise a group of low-molecular-weight proteins of approximately 7 kDa. CSP families that contain between two and nine members have been identified in food-related bacteria and several food-borne pathogens, including *Escherichia coli*, *Listeria monocytogenes*, *Staphylococcus aureus*, *Salmonella typhimurium* and *Pseudomonas fragi* [11]. In *E. coli*, the CSP family contains nine members (A-I), of which CspA is a well characterised RNA chaperone that reduces low temperature-associated increases in RNA secondary folding [10]. Although CSPs share a high degree of sequence similarity (>45%) with two conserved RNA-binding motifs, it is surprising that not all CSP members are cold-inducible, which implies that they may function in different cellular processes [11]. CspD in *E. coli* reportedly plays a negative regulatory role in chromosomal replication in nutrient-depleted cells [12]. Recent studies have indicated that the MqsR/MqsA toxin/antitoxin pair directly regulates CspD, which may be involved in toxicity and biofilm formation in *E. coli* [13].

Despite its significance in human health and in the aquaculture industry, the molecular mechanisms that underlie the low-temperature survival of *V. parahaemolyticus* remain largely unknown. Previous studies have revealed three *E. coli* CSP homologues in *V. parahaemolyticus*, including CspA, CspD and the cold shock DNA-binding domain-containing protein [14]. The *cspA* gene was up-regulated at the transcriptional level by over 30-fold after *V. parahaemolyticus* was treated for 60 min at 10 °C, a temperature below which bacterial growth was arrested [14]. However, the genes that encoded the other two homologues were undetectable by DNA microarray and real-time reverse transcription PCR (qRT-PCR) [14], which suggested that CspA could be a major CSP in *V. parahaemolyticus* during low-temperature growth. This study is the first to sequence, assemble and annotate the complete genome of *V. parahaemolyticus* CHN25 (serotype: O5:KUT), which has recently been isolated and characterised [15–17]. We constructed one dual-gene and two single-gene deletion mutants of the two main *V. parahemolyticus* CHN25 CSPs (designated as *Vpa*CspA and *Vpa*CspD) and determined the global-level gene expression profiles of the mutant and wild-type strains by Illumina RNA-Sequencing. These data will refine our grasp of the molecular mechanisms that underlie the low-temperature adaptation of the most common seafood-borne pathogen worldwide.

## Results and discussion

### Genomic features of *V. parahaemolyticus* CHN25

The complete genome sequence of *V. parahaemolyticus* CHN25 was determined by 454-pyrosequencing (see Methods). It consisted of two circular chromosomes that contained 3,416,467 bp and 1,843,316 bp (see Additional file 1: Figure S1). The genome also contained three plasmids (92,495 bp, 83,481 bp and 7,642 bp), all of which were absent from the other known *V. parahaemolyticus* genomes (see Additional file 2: Figure S2). The complete *V. parahaemolyticus* CHN25 genome contained 5,443,401bp with a 45.2% G + C content; 4,724 protein-encoding genes were predicted, of which approximately 34.8% encoded hypothetical proteins with unknown functions in public databases. Additionally, 9 rRNA operons and 55 ribosomal protein-encoding genes, 107 tRNA genes, and 30 pseudogenes were identified and annotated.

In marked contrast to the other known *V. parahaemolyticus* genomes, an integrative and conjugative element (ICE*Vpa*Chn1) was identified in the CHN25 genome. The 89.9-kb element (VpaChn25_2302 to Chn25_2378) contained sulfamethoxazole and streptomycin resistance genes. Mating assays demonstrated the active self-transmissibility of the antibiotic resistance from *V. parahaemolyticus* CHN25 to *E. coli* MG1655 [15]. Five prophage gene clusters that ranged from 6.5 to 36.6 kb were identified in the CHN25 genome, and they displayed high degrees of sequence identity with *Vibrio* phage martha 12B12 (GenBank accession no. HQ316581), *Vibrio* phage VPUSM 8 (GenBank accession no. KF361475), *Vibrio* phage henriette 12B8 (GenBank accession no. HQ316582), and *Vibrio* phage N4 [18]. Additionally, five insertion sequences (ISs) were detected in the genome, including ISShfr9 (Tn3), ISVal1, ISVpa3 (IS5) and ISVsa3 (IS91); the latter existed as two copies in the genome, which suggested that it was probably active. We concluded that the *V. parahaemolyticus* CHN25 genome has undergone major rearrangements due to its mobile genetic elements.

Consistent with the other *V. parahaemolyticus* genomes, most of the genes that encoded enzymes for the predicted central metabolic pathways were present in the CHN25 strain, including those required for glycolysis, oxidative phosphorylation and tricarboxylic acid cycle (TCA). Additionally, the CHN25 genome also contained genes for three restriction and modification (R-M) systems (types I, II and IV) and four DNA repair systems (base excision repair, nucleotide excision repair, mismatch repair and homologous recombination), most of which were present in several other *V. parahaemolyticus* strains. The high frequency of the horizontal gene transfer in the CHN25 strain (i.e., ICE*Vpa*Chn1) may have led the bacteria to hijack the R-M and DNA repair mechanisms to generate genetic diversity without losing genomic stability [19].

### Construction of the Δ*VpacspA*, Δ*VpacspD* and Δ*VpacspAD* mutants *of V. parahaemolyticus* CHN25

To investigate the low-temperature adaptation that was mediated by the predicted CSPs in *V. parahaemolyticus* CHN25, we constructed a deletion mutant of the *VpacspA* gene. The upstream and downstream sequences (approximately 0.5 kb) that flanked the *VpacspA* gene were obtained by PCR and cloned into a suicide vector, pDS132, to yield the recombinant vector, pDS132 + Δ*VpacspA*. The inserted 1,041-bp sequence was confirmed by DNA sequencing (data not shown). The recombinant vector was transformed into *E. coli* β2155, and the chloramphenicol-resistant transformant was obtained and conjugated with *V. parahaemolyticus* CHN25. Positive exconjugants were obtained using the two-step allelic exchange method (see the Methods section) and validated by PCR. DNA sequencing of the PCR product further confirmed the in-frame deletion of the 213-bp sequence of the *VpacspA* gene from the *V. parahaemolyticus* CHN25 genome (data not shown).

Similarly, the *VpacspD* gene that encoded a cold shock-like protein was deleted from the bacterial genome using the aforementioned method. The Δ*VpacspD* mutant with a 219-bp in-frame deletion was confirmed by DNA sequencing (data not shown). Furthermore, the *VpacspD* gene was also successfully deleted from the Δ*VpacspA* mutant, yielding a dual-gene deletion mutant of Δ*VpacspAD* (data not shown). The genome-level transcriptome data provided direct evidence of the successful construction of the three mutants, in which expression of the corresponding *VpacspA* or *VpacspD* genes was undetectable (see below).

### Survival of the Δ*VpacspA*, Δ*VpacspD* and Δ*VpacspAD* mutants at 10 °C

**Fig. 1:**
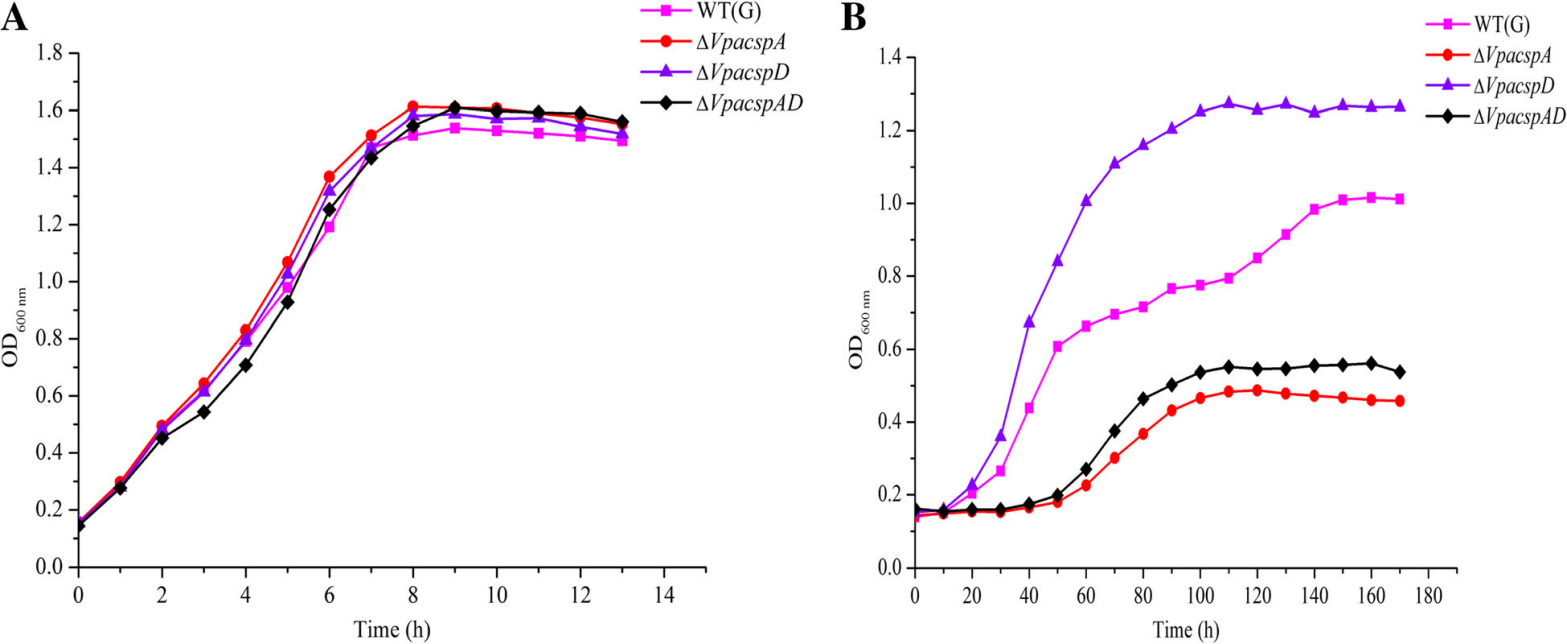
Growth of *V. parahaemolyticus* CHN25 and the Δ*VpacspA*, Δ*VpacspD* and Δ*VpacspAD* mutants in LB broth (3% NaCl, pH 8.5) at 37 °C (**a**) and 10 °C (**b**)

**Fig. 2:**
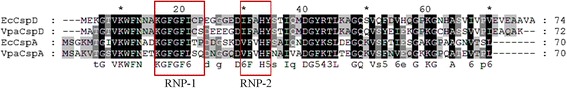
A multi-sequence alignment of the CSPs from *V. parahaemolyticus* CHN25 and *E. coli*. The numbers above the alignments indicate the relative positions of the entirely aligned sequences. Identical and conserved (>50% of the sequences) amino acid residues are highlighted in *black* and *grey*, respectively; the consensus sequence is shown below the alignment. The RNA-binding motifs (RNP-1 and RNP-2) are boxed. The EcCspA and EcCspD sequences were derived from *E. coli* JM83 (Yamanaka et al. [12]), while the *Vpa*CspA (VpaChn25A_0413) and *Vpa*CspD (VpaChn25_1036) sequences were obtained from *V. parahaemolyticus* CHN25 in this study

To gain insights into the possible effects of the CSP-associated gene deletions on *V. parahaemolyticus* CHN25 low-temperature survival, we determined growth curves for the Δ*VpacspA*, Δ*VpacspD* and Δ*VpacspAD* mutants, which were grown in LB broth (3% NaCl, pH 8.5) at 37 °C or 10 °C. As shown in Fig. 1 (A), no apparent differences in growth were observed between the mutant and wild-type strains at 37 °C, which was an optimal growth temperature. However, the Δ*VpacspA* mutant showed a longer lag phase (>30 h) and grew more slowly compared with the wild-type strain at 10 °C (Fig. 1b), demonstrating that *Vpa*CspA was a crucial CSP in *V. parahaemolyticus* CHN25 low-temperature survival. Although *Vpa*CspD was identified as one of the three homologues of the *E. coli* CSPs [14], the *VpacspD* gene deletion unexpectedly stimulated mutant growth at 10 °C in our study, which was notably faster than the wild-type strain (Fig. 1b), indicating that *Vpa*CspD likely functioned as a low-temperature bacterial growth inhibitor. A BLAST analysis revealed that the *Vpa*CspD sequence shared a 70% amino acid identity with CspD in *E. coli* (*Ec*CspD) (Fig. 2), which has been proposed to function as a novel inhibitor of DNA replication in nutrient-depleted cells [12]. Unlike *Ec*CspD, its null mutant grew well over a 15 to 42 °C temperature range with no detectable morphological changes. Our data indicated that *VpacspD* also functioned as a low-temperature induced-CSP (see below). Because only three CSP-associated genes were identified in *V. parahaemolyticus* and because *Vpa*CspD only displayed a 48% amino acid sequence identity with *Vpa*CspA (Fig. 2), *Vpa*CspD may have evolved to gain different biological functions. Interestingly, the Δ*VpacspAD* mutant also grew poorly at low temperature compared to the wild-type strain (Fig. 1b), indicating that the phenotype of the *VpacspA* gene deletion dominated that of the *VpacspD* gene (see below).

### Construction of the reverse mutants *ΔVpacspA*-com and *ΔVpacspD*-com and complementary phenotypes at 10 °C

**Fig. 3:**
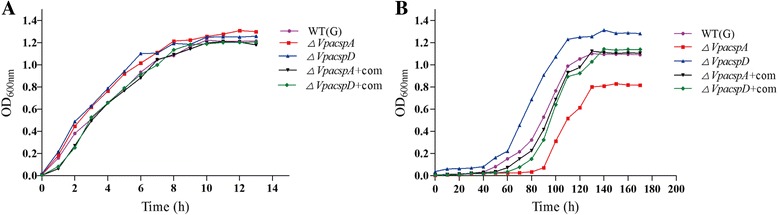
Growth of *V. parahaemolyticus* CHN25, the mutants (Δ*VpacspA*, Δ*VpacspD*), and the reverse mutants (Δ*VpacspA*-com, Δ*VpacspD*-com) at 37 °C (**a**) and 10 °C (**b**). The wild type and the mutants were incubated in LB broth (3% NaCl, pH 8.5), and the reverse mutants in the LB supplemented with 5 μg/mL chloramphenicol

The *cspA* gene was amplified from the genomic DNA of *V. parahaemolyticus* CHN25 by PCR, and cloned into the expression vector pMMB207, which yielded the recombinant vector pMMB207-*VpacspA*. The inserted 213-bp sequence was confirmed by DNA sequencing (data not shown). This recombinant vector was then electrotransformed into the *ΔVpacspA* mutant competent cells, and generated the reverse mutant *ΔVpacspA*-com (see the Methods section). Similarly, the recombinant vector pMMB207-*VpacspD* carrying the 240-bp *cspD* gene was also constructed, and electrotransformed into the *ΔVpacspD* mutant, yielding the reverse mutant *ΔVpacspD*-com*.* The growth curves for the reverse mutants *ΔVpacspA*-com and *ΔVpacspD*-com were also determined, which were incubated in LB broth (3% NaCl, 5 μg/mL chloramphenicol, pH 8.5) at 37 °C or 10 °C (Fig. 3). Consistent with the results in Fig. 1a, no obvious difference in growth at 37 °C was observed among the wild type, the mutants *ΔVpacspA* and *ΔVpacspD*, and the reverse mutants *ΔVpacspA*-com and *ΔVpacspD*-com (Fig. 3a). However, at 10 °C, the reverse mutants displayed similar growth phenotype as the wild type (Fig. 3b), demonstrating that the distinct phenotypes of the mutants *ΔVpacspA* and *ΔVpacspD* were indeed resulted from the *VpacspA* and *VpacspD* gene deletions in *V. parahaemolyticus* CHN25.

### Transcriptome profiles for the Δ*VpacspA,* Δ*VpacspD* and Δ*VpacspAD* mutants at 10 °C

To further investigate the *Vpa*CspA- and *Vpa*CspD-mediated low-temperature survival of *V. parahaemolyticus* CHN25, we determined global-level gene expression profiles for the Δ*VpacspA*, Δ*VpacspD* and Δ*VpacspAD* mutants that were grown at 10 °C, where distinct growth phenotypes were evident. Based on the complete genome sequence of *V. parahaemolyticus* CHN25, this analysis revealed numerous differentially expressed genes (DEGs) in the mutants, indicating that *Vpa*CspA and *Vpa*CspD likely functioned as master or global regulators in low-temperature bacterial growth. Five hundred seventy-two genes were significantly altered in the Δ*VpacspA* mutant compared with the wild-type strain; these genes represented approximately 12.4% of the expressed genes in *V. parahaemolyticus* CHN25. Of these, 263 genes showed higher transcriptional levels (fold change ≥ 2.0), while 309 genes were down-regulated (fold change ≤ 0.5). The altered genes in the Δ*VpacspA* mutant were grouped into eighty-three gene functional catalogues that were identified in the Kyoto Encyclopaedia of Genes and Genomes (KEGG) database (data not shown). The *VpacspD* gene deletion elicited 10% of the differentially expressed genes in the bacterium, including 242 up-regulated and 219 down-regulated genes that were grouped into seventy-six gene functional catalogues (data not shown). Additionally, the expression of 352 and 289 genes was up- and down-regulated, respectively, in the dual-gene deletion mutant (Δ*VpacspAD*), which accounted for 13.9% of the expressed genes; they were grouped into seventy-four gene functional catalogues (data not shown). A complete list of the DEGs for the three mutants is available in the Gene Expression Omnibus database (http://www.ncbi.nlm.nih.gov/geo/) under accession number GSE65998. To validate the transcriptome data, we examined ten representative genes for each of the three mutants by qRT-PCR. The resulting data were correlated with data from the Illumina RNA-Sequencing analysis, and there was no statistically significant difference between the two datasets (*P* = 0.982) (Table 1).

### The major low-temperature survival-associated metabolic pathways that were altered in the Δ*VpacspA*, Δ*VpacspD* and Δ*VpacspAD* mutants

#### Major altered metabolic pathways in the Δ*VpacspA* mutant

Based on the gene set enrichment analysis (GSEA) of the transcriptome data against the KEGG database, the following seven significantly altered metabolic pathways were identified in the Δ*VpacspA* mutant at 10 °C: valine, leucine and isoleucine degradation; the propanoate, ascorbate and aldarate, sulphur, and glycerophospholipid metabolic pathways; ATP-binding cassette (ABC) transporters; and bacterial secretion systems (Table 2). Of these, the DEGs that were linked to valine, leucine and isoleucine degradation as well as propanoate metabolism were up-regulated (2.1029- to 8.5787-fold), which may have resulted in increases in acetyl-CoA and propanoyl-CoA and subsequent entry into the TCA and pyruvate metabolic cycles, respectively, by the Δ*VpacspA* mutant.

**Table 1:** The qRT-PCR-validated DEGs in the transcriptome data

Locus / gene in *V. parahemolyticus* CHN25	Description of encoded protein	Fold change	
		RNA-Seq	RT-PCR
Δ*VpacspA* mutant			
VpaChn25A_0188	Hypothetical protein	13.41	10.21
VpaChn25_1642	Tricarboxylic transport TctC	10.04	10.35
VpaChn25_1640	GntR family transcriptional regulator	4.24	2.49
VpaChn25A_0561	Aldehyde dehydrogenase	7.05	5.24
VpaChn25_0068	Hyperosmotically inducible periplasmic protein	3.03	2.33
VpaChn25A_1398	L-threonine 3-dehydrogenase	0.07	0.03
VpaChn25A_1399	2-amino-3-ketobutyrate CoA ligase	0.07	0.03
VpaChn25_2988	Amino acid ABC transporter substrate-binding protein	0.14	0.09
VpaChn25A_0149	Transcriptional regulator CpxR	0.22	0.12
VpaChn25A_0568	Transcriptional regulator BetI	0.48	0.32
Δ*VpacspD* mutant			
VpaChn25_1716	Glycine betaine transporter periplasmic subunit	16.04	19.13
VpaChn25A_0188	Hypothetical protein	12.6	11.11
VpaChn25_1642	Tricarboxylic transport TctC	7.95	7.13
VpaChn25A_0561	Aldehyde dehydrogenase	4.08	3.23
VpaChn25_1640	GntR family transcriptional regulator	2.29	2.62
VpaChn25_2248	Glycerol uptake facilitator protein GlpF	0.04	0.02
VpaChn25A_1398	L-threonine 3-dehydrogenase	0.05	0.03
VpaChn25A_1399	2-amino-3-ketobutyrate CoA ligase	0.048	0.021
VpaChn25_2988	Amino acid ABC transporter substrate-binding protein	0.2	0.14
VpaChn25A_0149	Transcriptional regulator CpxR	0.3	0.25
Δ*VpacspAD* mutant			
VpaChn25A_1312	PTS system fructose-specific transporter subunit IIABC	8.77	21.54
VpaChn25A_0303	PTS system fructose-specific transporter subunit IIA	8.07	19.04
VpaChn25A_1313	Mannose-6-phosphate isomerase	5.17	16.82
VpaChn25_0669	Trehalose-6-phosphate hydrolase	4.51	9.77
VpaChn25A_0561	Aldehyde dehydrogenase	2.97	4.1
VpaChn25_2248	Glycerol uptake facilitator protein GlpF	0.033	0.039
VpaChn25_2249	Glycerol kinase	0.04	0.07
VpaChn25_0112	Phosphoenolpyruvate carboxykinase	0.17	0.22
VpaChn25_2988	Amino acid ABC transporter substrate-binding protein	0.31	0.43
VpaChn25A_0149	Transcriptional regulator CpxR	0.42	0.46

**Table 2:** Major altered metabolic pathways in the Δ*VpacspA*, Δ*VpacspD* and Δ*VpacspAD* mutants of *V. parahaemolyticus* CHN25 grown at the low temperature

Changed metabolic pathway	Locus / Gene^*^	Fold change	Description of encoded protein
Δ*VpacspA* mutant			
Valine, leucine and isoleucine degradation	VpaChn25A_0480	2.4983	Acetyl-CoA acetyltransferase
	VpaChn25A_0554	3.0843	Hydroxymethylglutaryl-CoA lyase
	VpaChn25A_0555	3.2361	Acyl-CoA carboxylase alpha chain
	VpaChn25A_0556	2.4095	Enoyl-CoA hydratase/isomerase
	VpaChn25A_0557	2.662	Acyl-CoA carboxyltransferase beta chain
	VpaChn25A_0558	2.216	Acyl-CoA dehydrogenase
	VpaChn25A_0560	2.8734	Acyl-CoA thiolase
	VpaChn25A_0561	7.0543	Aldehyde dehydrogenase
	VpaChn25A_0565	7.3736	3-hydroxyisobutyrate dehydrogenase
	VpaChn25A_1033	2.7479	Methylmalonate-semialdehyde dehydrogenase
	VpaChn25A_1036	2.1029	Acyl-CoA dehydrogenase
Propanoate metabolism	VpaChn25_1376	6.0834	4-aminobutyrate aminotransferase
	VpaChn25_1635	6.3526	PrpE protein
	VpaChn25_1638	8.5787	Methylcitrate synthase
	VpaChn25_1639	6.7065	2-methylisocitrate lyase
	VpaChn25_2798	3.959	Acetyl-CoA synthetase
ABC transporters	VpaChn25A_0128	0.4376	ABC transporter substrate-binding protein
	VpaChn25A_0130	0.428	ABC transporter ATP-binding protein
	VpaChn25A_0533	3.1521	High-affinity branched-chain amino acid transport ATP-binding protein
	VpaChn25A_0535	3.9903	ABC transporter membrane spanning protein
	VpaChn25A_0536	5.0642	High-affinity branched-chain amino acid transport permease
	VpaChn25A_0537	6.8795	Hypothetical protein
	VpaChn25A_0538	4.7093	High-affinity branched-chain amino acid transport ATP-binding protein
	VpaChn25A_0571	0.3015	Glycine betaine-binding ABC transporter
	VpaChn25A_0572	0.3358	Permease
	VpaChn25A_0573	0.3801	ABC transporter ATP-binding protein
	VpaChn25A_0595	0.4067	Ribose ABC transporter permease
	VpaChn25A_0604	0.406	Iron (III) ABC transporter periplasmic iron-compound-binding protein
	VpaChn25A_1325	0.2076	Iron (III) ABC transporter ATP-binding protein
	VpaChn25A_1326	0.2262	Iron (III) ABC transporter periplasmic iron-compound-binding protein
	VpaChn25A_1327	0.3019	Iron-hydroxamate transporter permease subunit
	VpaChn25A_1333	2.1634	Transport protein
	VpaChn25A_1544	0.2026	Iron-dicitrate transporter substrate-binding subunit
	VpaChn25_0306	0.4761	Thiamine transporter membrane protein
	VpaChn25_0363	2.1743	ABC transporter substrate binding protein
	VpaChn25_0846	0.4129	Zinc ABC transporter permease
	VpaChn25_0848	0.4556	Zinc ABC transporter periplasmic zinc-binding protein
	VpaChn25_1344	0.2076	Oligopeptide ABC transporter ATP-binding protein
	VpaChn25_1345	0.2716	Oligopeptide ABC transporter ATP-binding protein
	VpaChn25_1346	0.2355	Oligopeptide ABC transporter permease
	VpaChn25_1347	0.3245	Oligopeptide ABC transporter permease
	VpaChn25_1348	0.3723	Oligopeptide ABC transporter periplasmic oligopeptide-binding protein
	VpaChn25_1613	2.4584	Amino acid ABC transporter substrate-binding protein
	VpaChn25_1714	0.4917	Glycine/betaine/proline ABC transporter
	VpaChn25_1913	2.222	Hypothetical protein
	VpaChn25_2428	0.4529	Iron (III) ABC transporter permease
	VpaChn25_2429	0.3234	Iron (III) ABC transporter periplasmic iron-compound-binding protein
	VpaChn25_2987	0.2357	Amino acid ABC transporter permease
	VpaChn25_2988	0.1424	Amino acid ABC transporter substrate-binding protein
Bacterial secretion systems	VpaChn25A_0952	4.5459	Hypothetical protein
	VpaChn25A_0954	6.1398	ClpA / B-type chaperone
	VpaChn25A_0966	6.0405	Hypothetical protein
	VpaChn25A_0967	7.0563	Hypothetical protein
	VpaChn25A_0969	2.4559	Hypothetical protein
	VpaChn25_1662	0.3404	Type III secretion system protein
	VpaChn25_1663	0.2481	Type III secretion system protein
	VpaChn25_1664	0.2099	Translocation protein in type III secretion
	VpaChn25_1665	0.2746	Translocation protein in type III secretion
	VpaChn25_1666	0.2109	Translocation protein in type III secretion
	VpaChn25_1887	0.4446	Outer membrane protein TolC
Ascorbate and aldarate metabolism	VpaChn25A_0230	0.1973	PTS system ascorbate-specific transporter subunit IIC
	VpaChn25A_0231	0.1668	Sugar phosphotransferase component II B
	VpaChn25A_0232	0.1096	Phosphotransferase enzyme II, A component
Sulfur metabolism	VpaChn25_0788	0.2523	Cysteine synthase A
	VpaChn25_0937	3.0238	Cysteine synthase / cystathionine beta-synthase family protein
	VpaChn25_1397	2.7136	Homoserine O-succinyltransferase
	VpaChn25_2650	0.4424	Phosphoadenosine phosphosulfate reductase
	VpaChn25_2651	0.4934	Sulfite reductase subunit beta
	VpaChn25_2652	0.2017	Sulfite reductase (NADPH) flavoprotein subunit alpha
	VpaChn25_2692	2.1317	Cystathionine gamma-synthase
Glycerophospholipid metabolism	VpaChn25A_0425	0.4957	Diacylglycerol kinase
	VpaChn25A_0732	2.2688	Outer membrane phospholipase A
	VpaChn25_0642	0.4375	Phosphatidylglycerophosphatase A
	VpaChn25_0885	0.3795	Surfactin synthetase
	VpaChn25_2245	0.2175	Glycerophosphodiester phosphodiesterase
	VpaChn25_2251	0.2013	Glycerol-3-phosphate dehydrogenase
Δ*VpacspD* mutant			
PTS	VpaChn25A_0230	0.2003	PTS system ascorbate-specific transporter subunit IIC
	VpaChn25A_0231	0.1886	Sugar phosphotransferase component II B
	VpaChn25A_0232	0.1253	Phosphotransferase enzyme II, A component
	VpaChn25A_0303	12.8135	PTS system fructose-specific transporter subunit IIA
	VpaChn25A_1196	0.3193	Mannitol-specific PTS system enzyme II component
	VpaChn25A_1309	3.4141	PTS system fructose-specific transporter subunit IIB
	VpaChn25A_1312	4.9223	PTS system fructose-specific transporter subunit IIABC
	VpaChn25_0668	0.4181	PTS system trehalose (maltose)-specific transporter subunits IIBC
	VpaChn25_2564	2.1655	PTS system cellobiose-specific transporter subunit IIA
	VpaChn25_2566	2.8755	PTS system cellobiose-specific transporter subunit IIB
Alanine, aspartate and glutamate metabolism	VpaChn25A_0370	3.4733	Adenylosuccinate synthase
	VpaChn25_0104	2.2225	Glutamine synthetase
	VpaChn25_0345	0.3501	Glucosamine-fructose-6-phosphate aminotransferase
	VpaChn25_0436	2.1782	Glutamate synthase subunit beta
	VpaChn25_0437	2.0222	Glutamate synthase subunit alpha
	VpaChn25_1375	3.4554	Succinate-semialdehyde dehydrogenase
	VpaChn25_1376	3.0672	4-aminobutyrate aminotransferase
	VpaChn25_2552	0.4804	Glutaminase
	VpaChn25_2583	2.0986	Aspartate carbamoyltransferase
	VpaChn25_2584	2.7419	Aspartate carbamoyltransferase
	VpaChn25_2784	0.4744	Aspartate ammonia-lyase
Arginine and proline metabolism	VpaChn25_1371	2.2205	Aldehyde dehydrogenase
	VpaChn25_1372	2.779	Oxidoreductase
	VpaChn25_1373	3.3592	Carbon-nitrogen hydrolase
	VpaChn25_1374	3.206	Hypothetical protein
	VpaChn25_2581	3.3917	Arginine deiminase
	VpaChn25_2719	2.8035	Succinylglutamic semialdehyde dehydrogenase
	VpaChn25_2720	3.9099	Arginine/ornithine succinyltransferase
	VpaChn25_2721	2.1486	Bifunctional N-succinyldiaminopimelate-aminotransferase/acetylornithine transaminase protein
Valine, leucine and isoleucine degradation	VpaChn25A_0480	3.4643	Acetyl-CoA acetyltransferase
	VpaChn25A_0555	2.023	Acyl-CoA carboxylase alpha chain
	VpaChn25A_0557	2.0591	Acyl-CoA carboxyltransferase beta chain
	VpaChn25A_0561	4.081	Aldehyde dehydrogenase
	VpaChn25A_0565	3.2953	3-hydroxyisobutyrate dehydrogenase
	VpaChn25A_1038	0.4859	Enoyl-CoA hydratase / isomerase
	VpaChn25_0020	2.6571	Multifunctional fatty acid oxidation complex subunit alpha
	VpaChn25_2076	0.4816	3-ketoacyl-CoA thiolase
Propanoate metabolism	VpaChn25_1635	2.316	PrpE protein
	VpaChn25_1638	4.6552	Methylcitrate synthase
	VpaChn25_1639	3.654	2-methylisocitrate lyase
	VpaChn25_2433	3.0153	Bifunctional aconitate hydratase 2/2-methylisocitrate dehydratase
	VpaChn25_2798	2.1035	Acetyl-CoA synthetase
Nitrogen metabolism	VpaChn25A_0296	4.3124	Oxidoreductase protein
	VpaChn25A_0917	3.7442	Nitrite reductase large subunit
	VpaChn25A_1392	0.4447	Carbonic anhydrase
	VpaChn25_1817	9.4056	Cytochrome c552
	VpaChn25_2448	0.2912	Carbonic anhydrase
Δ*VpacspAD* mutant			
TCA	VpaChn25_0313	0.2603	Malate dehydrogenase
	VpaChn25_0837	0.1445	Type II citrate synthase
	VpaChn25_0838	0.2716	Succinate dehydrogenase cytochrome b556 large membrane subunit
	VpaChn25_0840	0.402	Succinate dehydrogenase flavoprotein subunit
	VpaChn25_0841	0.3554	Succinate dehydrogenase iron-sulfur subunit
	VpaChn25_0843	0.3915	Dihydrolipoamide succinyltransferase
	VpaChn25_0844	0.3535	Succinyl-CoA synthetase subunit beta
	VpaChn25_0845	0.3009	Succinyl-CoA synthetase subunit alpha
	VpaChn25_1035	0.4921	Isocitrate dehydrogenase
	VpaChn25_2762	0.478	Fumarate reductase flavoprotein subunit
	VpaChn25_2763	0.4836	Fumarate reductase iron-sulfur subunit
	VpaChn25_2764	0.4898	Fumarate reductase subunit C
	VpaChn25_2765	0.3058	Fumarate reductase subunit D
PTS	VpaChn25A_0230	0.3036	PTS system ascorbate-specific transporter subunit IIC
	VpaChn25A_0231	0.2665	Sugar phosphotransferase component II B
	VpaChn25A_0232	0.232	Phosphotransferase enzyme II, A component
	VpaChn25A_0303	8.0738	PTS system fructose-specific transporter subunit IIA
	VpaChn25A_1312	8.7743	PTS system fructose-specific transporter subunit IIABC
	VpaChn25_0356	0.3818	PTS system mannitol-specific transporter subunit IIABC
	VpaChn25_2566	0.3343	PTS system cellobiose-specific transporter subunit IIB
	VpaChn25_0668	3.948	PTS system trehalose (maltose)-specific transporter subunits IIBC
Butanoate metabolism	VpaChn25A_0528	2.6111	Acetoacetyl-CoA synthetase
	VpaChn25A_0560	2.8181	Acyl-CoA thiolase
Fructose and mannose metabolism	VpaChn25A_1313	5.1663	Mannose-6-phosphate isomerase
	VpaChn25_0355	0.3626	Mannitol-1-phosphate 5-dehydrogenase
Pyruvate metabolism	VpaChn25A_0307	2.3933	Hypothetical protein
	VpaChn25A_0367	0.321	Phosphoenolpyruvate synthase
	VpaChn25A_0934	0.4136	D-lactate dehydrogenase
	VpaChn25_0112	0.1659	Phosphoenolpyruvate carboxykinase
	VpaChn25_1693	2.2321	Aldehyde dehydrogenase
	VpaChn25_1927	0.408	Pyruvate kinase II
Oxidative phosphorylation	VpaChn25A_0546	2.1237	Cytochrome BD2 subunit II
	VpaChn25_1519	0.4848	Cytochrome c oxidase subunit CcoP
	VpaChn25_1521	0.4651	cbb3-type cytochrome c oxidase subunit II
Cysteine and methionine metabolism	VpaChn25_0576	0.293	Homocysteine synthase
	VpaChn25_0788	0.2948	Cysteine synthase A
	VpaChn25_0937	2.3269	Cysteine synthase/cystathionine beta-synthase family protein
	VpaChn25_1880	2.8826	5-methyltetrahydropteroyltriglutamate-homocysteine S-methyltransferase
	VpaChn25_2471	0.4433	S-ribosylhomocysteinase
	VpaChn25_2646	2.2961	Aspartate kinase
Arginine and proline metabolism	VpaChn25A_1611	0.4145	Bifunctional proline dehydrogenase/pyrroline-5-carboxylate dehydrogenase
	VpaChn25_1332	0.1954	Hydroxyproline-2-epimerase
	VpaChn25_1335	0.3527	Ornithine cyclodeaminase
	VpaChn25_1544	0.3291	NAD-glutamate dehydrogenase
	VpaChn25_2719	0.3969	Succinylglutamic semialdehyde dehydrogenase
	VpaChn25_2720	0.482	Arginine /ornithine succinyltransferase
	VpaChn25_2721	0.2687	Bifunctional N-succinyldiaminopimelate-aminotransferase / acetylornithine transaminase protein
Alanine, aspartate and glutamate metabolism	VpaChn25_0345	0.2743	Glucosamine-fructose-6-phosphate aminotransferase
	VpaChn25_0438	2.461	Glutamate synthase subunit beta
	VpaChn25_0439	2.6963	Glutamate synthase, large subunit
	VpaChn25_1114	0.407	Alanine dehydrogenase
	VpaChn25_2022	0.4771	Cytoplasmic asparaginase I

^*^VpaChn25, chromosome 1; VpaChn25A, chromosome 2

For the other five altered metabolic pathways, most of the DEGs were down-regulated in the Δ*VpacspA* mutant, which was directly related to its remarkable low-temperature growth inhibition. For example, the expression of twenty-four genes that were linked to ABC transporters was reduced (0.4917- to 0.1424-fold); they included the glycine betaine (GB)/proline, oligopeptide, iron(III) and zinc ABC transporters. This indicated the positive regulation of these ABC transporters by *Vpa*CspA during low-temperature *V. parahaemolyticus* CHN25 survival.

Bacterial secretion systems play important roles in virulence, symbiosis, interbacterial interactions, and environmental stress [20]. The genes that encoded components of the four secretion system types (T1SS, T2SS, T3SS1 and T6SS2) were identified in the *V. parahaemolyticus* CHN25 genome. Of these, eleven genes were differentially expressed in the Δ*VpacspA* mutant at the low temperature. Activation of the *tolC* gene, which encodes an outer membrane protein of T1SS, has been reported in *Psychrobacter cryohalolentis* K5 during growth at sub-zero temperatures [21]. In this study, *tolC* gene expression (VpaChn25_1887) was down-regulated (0.4446-fold) in the Δ*VpacspA* mutant, indicating that *Vpa*CspA positively regulated low-temperature *tolC* gene expression in *V. parahaemolyticus* CHN25. Likewise, the *yscQRSTU* genes (VpaChn25A_0952, 0954, 0966, 0967 and 0969), which encode the components of T3SS1, were also highly down-regulated (0.3404- to 0.2109-fold). However, the expression of five genes that were required for T6SS2 was strongly enhanced (2.4559- to 7.0563-fold) in the Δ*VpacspA* mutant, which was inconsistent with previous speculation [22]. Future investigations into the biological significance of the secretion systems and their differential expression characteristics during low-temperature survival by *V. parahaemolyticus* will provide important insights on this topic.

### Major altered metabolic pathways in the Δ*VpacspD* mutant

Based on the GESA-KEGG analysis, the following six significantly altered metabolic pathways were identified in the Δ*VpacspD* mutant at 10 °C: the phosphotransferase system (PTS); alanine, aspartate and glutamate metabolism; arginine and proline metabolism; the propanoate and nitrogen metabolic pathways; valine, leucine and isoleucine degradation.

Consistent with its active low-temperature growth phenotype, several DEGs that were linked to PTS, to nitrogen, arginine and proline metabolism and to alanine, aspartate and glutamate metabolism were significantly up-regulated in the Δ*VpacspD* mutant. A major barrier to protein function at low temperatures is the inability to maintain sufficient flexibility so that it can increase its interactions with substrates to reduce its required activation energy [23]. In arginine and proline metabolism, all eight DEGs were up-regulated in the Δ*VpacspD* mutant. For example, expression of an arginine deiminase (VpaChn25_2581) and an arginine/ornithine succinyltransferase (VpaChn25_2720), which are required to convert L-arginine to L-citrulline and then to N2-succinyl-L-arginine, were up-regulated by 3.3917- and 3.9099-fold, respectively. Arginines are structurally stabilizing factors that contain side chains that form salt bridges and hydrogen bonds [24]. Our data indicated that a low-temperature decrease in L-arginine in the Δ*VpacspD* mutant may have promoted increased protein flexibility. Moreover, the abundance of proline residues is related to increased protein stability due to the rigidity of the N-Cα bond [23]. In this study, a decrease in proline resulted from up-regulated proline metabolism-associated enzymes may have also enhanced protein flexibility in the Δ*VpacspD* mutant. To our knowledge, these genes have not been previously linked to low-temperature survival.

Expression of a glutamine synthetase (VpaChn25_0104), which catalyses L-glutamate to L-glutamine, was up-regulated in the alanine, aspartate and glutamate metabolic pathways. However, the genes that encoded a glutaminase (VpaChn25_2552) and a glucosamine-fructose-6-phosphate aminotransferase (VpaChn25_0345), which convert L-glutamine to L-glutamate and then to D-glucosamine, showed opposite expression profiles, which suggested a decrease in L-glutamate accumulation in the Δ*VpacspD* mutant. This was also suppressed in the psycrophilic proteins of *Vibrio salmonicida* [25].

Unexpectedly, the comparative transcriptome analysis revealed very few genes that were up-regulated in Δ*VpacspD* but down-regulated in the Δ*VpacspA* mutant, indicating that these genes were specifically and negatively governed by *Vpa*CspD. Additionally, in the Δ*VpacspA* mutant, *VpacspD* gene expression was increased (2.5073-fold) at the low temperature, which was validated by qRT-PCR analysis, but no significant change in *VpacspA* gene expression was observed in the Δ*VpacspD* mutant. The results indicated that *Vpa*CspD was inhibited by *Vpa*CspA at low temperatures, which was consistent with the growth phenotypes described above.

### Major altered metabolic pathways in the Δ*VpacspAD* mutant

Similarly, the GESA-KEGG analysis revealed the following nine significantly changed metabolic pathways in the Δ*VpacspAD* mutant at 10 °C: TCA; PTS; butanoate metabolism; fructose and mannose metabolism; the pyruvate and the cysteine and methionine metabolic pathways; arginine and proline metabolism; alanine, aspartate and glutamate metabolism; oxidative phosphorylation. Interestingly, these altered metabolic pathways were different from those that were induced in the Δ*VpacspA* mutant, although both mutants demonstrated the slower-growth phenotype at the low temperature. Most of the DEGs that were linked to TCA, oxidative phosphorylation, and pyruvate metabolism were inhibited in the Δ*VpacspAD* mutant, which may explain its slower growth at this low temperature. The down-regulated central metabolic pathways were also observed in other bacteria that were grown at a low temperature [26].

Similar to the Δ*VpacspD* mutant, the alanine, aspartate and glutamate metabolic pathways, PTS, and the arginine and proline metabolic pathways were also significantly changed in the Δ*VpacspAD* mutant. However, distinct expression patterns were detected in the two mutants. For example, in contrast to the Δ*VpacspD* mutant, all seven DEGs that were involved in arginine and proline metabolism were down-regulated (0.482- to 0.1954-fold) in the Δ*VpacspAD* mutant. Additionally, the phosphoenolpyruvate-dependent PTS is a major sugar transport multicomponent system in bacteria, by which multiple sugars are transported into bacteria, concomitantly phosphorylated, and fed into glycolysis [27]. In this study, expression of the genes that encoded the cellobiose- and trehalose (maltose)-specific transporter subunits (VpaChn25_2566 and Chn25_0668) also displayed opposite patterns between the Δ*VpacspAD* and Δ*VpacspD* mutants. These results highlighted the antagonistic regulatory effects by *Vpa*CspA and *Vpa*CspD on low-temperature survival of *V. parahaemolyticus* CHN25.

In cysteine and methionine metabolism, a homocysteine synthase (VpaChn25_0576) and S-ribosylhomocysteinase (VpaChn25_2471), which are involved in converting O-acetyl-L-homoserine and S-ribosyl-L-homocysteine to L-homocysteine, were down-regulated (0.2930- and 0.4433-fold, respectively) in the Δ*VpacspAD* mutant. However, a 5-methyltetrahydropteroyltriglutamate-homocysteine S-methyltransferase (VpaChn25_1880) that catalyses L-homocysteine to L-methionine was up-regulated (2.8826-fold). These results suggest the attenuation of L-homocysteine in the Δ*VpacspAD* mutant, which may reduce interference by L-homocysteine with amino acid metabolic and translation processes at low temperatures [28].

### Differentially expressed regulators (DERs) that are involved in the low-temperature survival of the Δ*VpacspA*, Δ*VpacspD* and Δ*VpacspAD* mutants

The *V. parahaemolyticus* CHN25 genome contains approximately two hundred and seventy-two genes that encode putative transcriptional or response regulators, which represent approximately 5.8% of all protein-encoding genes in the bacterium. Changes in the expression of transcription factors, especially the master regulators, can modulate global regulatory networks that, in some cases, are essential for bacterial adaptation to changing environments [13]. In this study, the genome-level transcriptome data revealed thirty, twenty-three and thirty-six DERs in the Δ*VpacspA*, Δ*VpacspD* and Δ*VpacspAD* mutants at 10 °C, respectively (see Additional file 3: Table S1). They globally or specifically regulate various cellular processes, including cold-temperature survival in bacteria, by regulating transcriptional or response regulators that are involved in DNA-binding, LysR-type transcriptional regulators, and GntR, AraC/XylS, ArsR, LuxR, and DeoR regulator families.

Of these regulators, several directly regulate gene expression in response to environmental signals in other bacteria. For example, a recombination regulator, RecX (VpaChn25_2483), which regulates DNA recombination and protects the cell from ionising radiation and UV-irradiation in *E. coli* [29], was notably down-regulated (0.2654-fold) in the Δ*VpacspA* mutant; this indicates the positive regulation of RecX by *Vpa*CspA in *V. parahaemolyticus* CHN25 at low temperatures. Interestingly, a transcriptional regulator, BetI (VpaChn25A_0568), was also inhibited in the Δ*VpacspA* mutant (0.4784-fold), which negatively regulated the *betT* and *betIBA* genes that governed GB synthesis from choline in *E. coli* [30]. Moreover, two genes (VpaChn25_1793 and Chn25_1442), which encode the osmotically inducible betaine-choline-carnitine transporters (BCCTs) that mediate the acquisition of preformed GB [31], were also down-regulated in the Δ*VpacspA* mutant. These data indicate that *Vpa*CspA may stimulate an accumulation of cellular GB that adjusts the hydration level of the bacterial cell cytoplasm at low temperatures [32, 33]. Additionally, expression of an important transcriptional regulator, PdhR (VpaChn25_2454), which belongs to the GntR family of transcriptional regulators, was repressed (0.3122-fold) in the Δ*VpacspA* mutant. PdhR regulates central metabolism by controlling transcription of the components that form the pyruvate dehydrogenase complex [34].

Among the DERs that were elicited in the Δ*VpacspD* mutant, two regulators (a response regulator (VpaChn25_1251) and a MerR family transcriptional regulator (VpaChn25A_1361)) were up-regulated in the Δ*VpacspD* mutant at low temperatures (2.891- and 2.8939-fold, respectively). The latter regulates gene transcription in response to different environmental signals, including signals from heavy metal ions, organic compounds, and oxidative stress [35]. Approximately 56.5% of the DERs in the Δ*VpacspD* mutant were down-regulated, of which half were exclusively expressed in the Δ*VpacspD* mutant (e.g., the two-component response regulator (VpaChn25A_1000) and sigma-E factor negative regulatory protein RseA (VpaChn25_2510)) [36].

Transcriptome data comparisons revealed mosaic DER expression profiles in the *VpacspAD* mutant. Interestingly, three regulators of T3SS1 gene expression were inhibited in both Δ*VpacspD* and Δ*VpacspAD* cells at the low temperature. These included ExsA (VpaChn25_1689) and ExsE (VpaChn25_1692), which belonged to the ExsACDE regulatory cascade, and a T3SS1 regulator (VpaChn25_1651), which was indicative of positive regulation of T3SS1 by *Vpa*CspD at low temperatures; this function was similar to that of *Vpa*CspA. Likewise, expression of the UhpC regulator (VpaChn25A_0772), a membrane-bound sensor for external glucose-6-phosphate in *E. coli* [37], was also decreased in the two mutants. UhpC was reported to negatively modulate a YE0480 gene in *Yersinia enterocolitica*, which encoded a homologue of the FhaC accessory protein; FhaC was strongly expressed at 10 °C but not at 37 °C in *Bordetella pertussis* [38].

Interestingly, three DERs were detected in all three mutants, and the other five were synchronously induced in both Δ*VpacspA* and Δ*VpacspD* cells, indicating either similar regulatory functions that were shared between *Vpa*CspA and *Vpa*CspD or *Vpa*CspA/D-independent regulation in *V. parahaemolyticus* CHN25 at low temperatures. The molecular responses of bacteria to external environmental signals are complex, but two-component signal transduction systems reportedly play important roles in low-temperature adaptation by several bacteria [39–41]. In this study, the expression of a cytosolic response regulator, CpxR (VpaChn25A_0149), which belongs to the two-component Cpx-envelope stress system [42], was repressed in the three mutants. The Cpx system responds to a broad range of environmental stimuli (e.g., pH, salt, metals, lipids and misfolded proteins) that cause perturbation of the envelope [43]. In this study, our data showed positive regulation of CpxR by both *Vpa*CspA and *Vpa*CspD, which may have protected envelope-bound proteins from low-temperature damage.

Taken together, our transcriptome data revealed a complex molecular regulatory network that was specifically, coordinately or antagonistically modulated by *Vpa*CspA and *Vpa*CspD during low-temperature adaptation by *V. parahaemolyticus*. Numerous regulators, which act as activators or repressors in response to multiple environmental stressors in bacteria, were also elicited in the three mutants. A future in-depth regulatory network analysis will improve our understanding of low-temperature adaptation mechanisms in *V. parahaemolyticus*.

### Possible low-temperature adaptation mechanisms that are mediated by *Vpa*CspA and *Vpa*CspD in *V. parahaemolyticus* CHN25

**Fig. 4:**
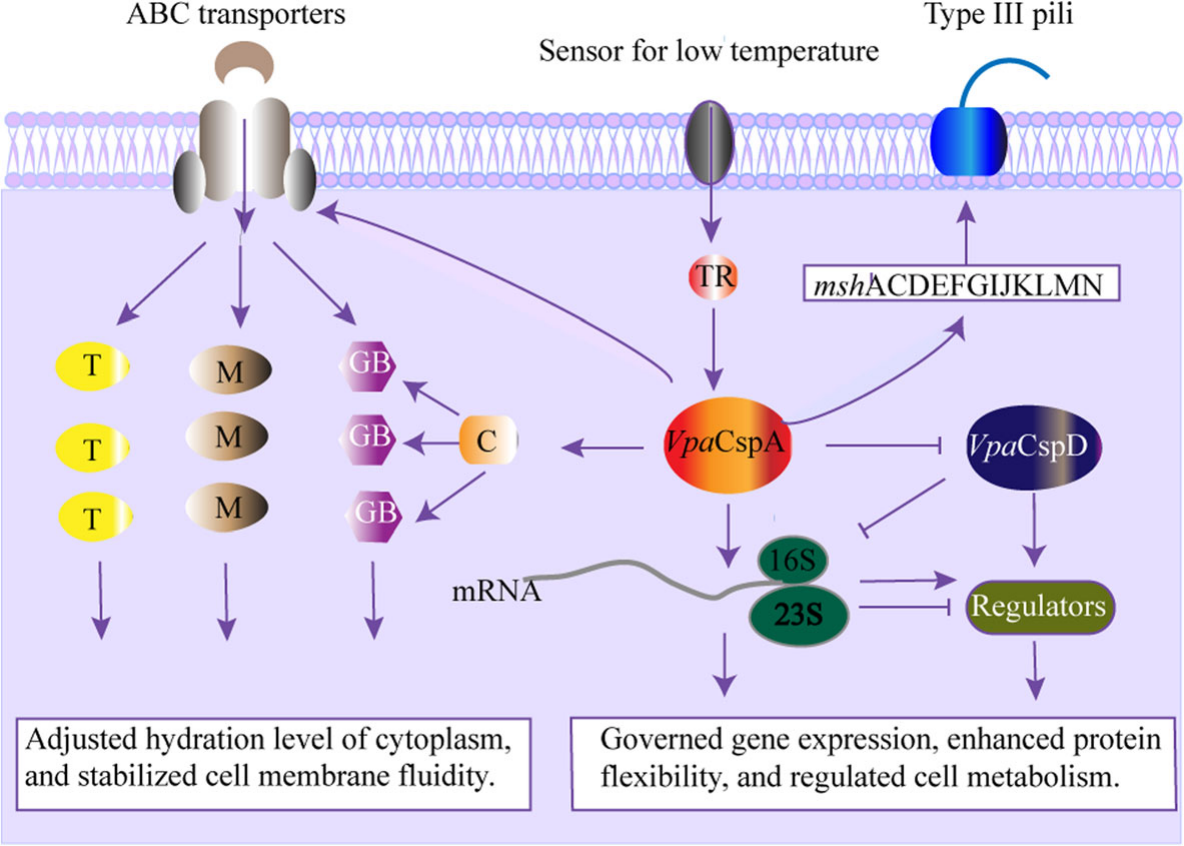
The possible *Vpa*CspA and *Vpa*CspD-mediated molecular mechanisms that underlie low-temperature adaptation by *V. parahaemolyticus* CHN25. T, trehalose; M, mannitol; GB, glycine betaine; C, cAMP regulator protein; TR, transducer; 16S and 23S, rRNA subunits

The most common strategy that has been adopted by bacteria to survive a low-temperature environment is the accumulation of compatible solutes (e.g., GB, choline, carnitine, and mannitol) by uptake or biosynthesis [11]. In this study, a similar low-temperature strategy by *V. parahaemolyticus* CHN25 was observed (Fig. 4). For example, seven genes that were associated with GB biosynthesis, BCCT and GB-binding ABC transporters were significantly inhibited in the Δ*VpacspA* mutant, which indicated that *Vpa*CspA likely stimulated cellular GB accumulation to adjust the hydration level of the cytoplasm and to protect the bacterium from low-temperature damage.

Interestingly, in this study, the glycerophospholipid metabolism-associated *glpDFKQ* genes were more strongly inhibited in Δ*VpacspAD* than in the Δ*VpacspA* or Δ*VpacspD* mutants, which indicated a coordinated low-temperature activation of the genes by *Vpa*CspA and *Vpa*CspD. For example, expression of the *glpF* gene (*VpaChn25_2248*), which encodes a glycerol uptake facilitator and functions in substrate equilibration between the extracellular and intracellular spaces [44], was down-regulated in Δ*VpacspA* (0.2794-fold), strongly suppressed in Δ*VpacspD* (0.0457-fold), and suppressed in Δ*VpacspAD* (0.0337-fold). Similarly, the *glpQ* and *glpD* genes encode a glycerophosphodiester phosphodiesterase (VpaChn25_2245) and a glycerol-3-phosphate dehydrogenase (VpaChn25_2251), and they catalyse sn-glycero-3-phosphocholine to choline and sn-glycerol-3-phosphate (G3P) and G3P to dihydroxyacetone phosphate (DHAP), respectively. Expression of the *glpQ* and *glpD* genes was also more strongly inhibited in Δ*VpacspAD* (0.0787- and 0.0636-fold, respectively) than in Δ*VpacspA* (0.2175- and 0.2013-fold, respectively) or Δ*VpacspD* (0.1011- and 0.0302-fold, respectively), indicating a positively superposed regulation of the choline biosynthesis genes by *Vpa*CspA and *Vpa*CspD; this may have resulted in an increase in cellular compatible solutes to maintain cell membrane integrity at low temperatures. However, the decreased DHAP indirectly led to increased biofilm formation and contributed to several survival advantages under various environmental and energy insults in several other bacteria [45, 46]. Moreover, the *glpK* gene, which encodes a glycerol kinase (VpaChn25_2249) that catalyses glycerol to G3P, showed similar expression profiles in all three mutants, which probably resulted in attenuated cellular G3P accumulation at low temperatures. G3P has been reported to mediate catabolite repression through adenylate cyclase inhibition, which leads to decreases in 3’-5’-cyclic adenosine monophosphate (cAMP) and inactivation of the cAMP receptor protein (CRP); CRP is a global regulator that participates in sugar metabolism and plays an important role in cold adaptation by *E. coli* [44].

Protective roles for trehalose in response to low-temperature, heat and osmotic stressors have been reported, including prevention of the denaturation and aggregation of specific proteins, in vivo activity as a free radical scavenger, and stabilisation of cell membrane fluidity [47]. In this study, expression of the trehalose (maltose)-specific transporter subunit II BC components (VpaChn25_0668) was down-regulated in the Δ*VpacspA* (0.2935-fold) and Δ*VpacspD* (0.4181-fold) mutants, indicating the positive regulation of trehalose-specific transport by *Vpa*CspA and *Vpa*Csp*D* to promote bacterial adaptation to a low-temperature environment. Nevertheless, the gene showed an opposite expression pattern in the Δ*VpacspAD* mutant (3.948-fold), which implied unknown regulatory mechanisms in the Δ*VpacspAD* mutant by which trehalose was transported.

Biofilm formation is related to bacterial survival in various environments. It has been reported that type IV pili (TFP) played an important role in the biofilm formation of *V. parahaemolyticus* [48]. In this study, the complete genome sequence analysis revealed a mannose-sensitive hemagglutinin gene cluster (*mshACDEFGIJKLMN*) that was required for TFP formation in *V. parahaemolyticus* CHN25. Interestingly, the *msh* gene cluster was significantly down-regulated in the Δ*VpacspA* mutant, which indicated a positive regulation of TFP by *Vpa*CspA. The enhanced biofilm formation likely increased the persistence of *V. parahaemolyticus* in the aquatic environment by enhancing low-temperature colonisation of environmental surfaces [49].

Additionally, our transcriptome data also revealed several other molecular mechanisms that facilitated the low-temperature survival of *V. parahaemolyticus* CHN25 (Fig. 4). For example, *Vpa*CspD negatively regulated arginine and proline metabolism, which likely resulted in increased cellular protein flexibility and stability so that efficient functionality could be maintained at the low temperature.

## Conclusions

This study is the first to describe the complete 5,443,401-bp genome sequence (45.2% G + C) of *V. parahaemolyticus* CHN25 (serotype: O5:KUT), which consists of two circular chromosomes and three plasmids with 4,724 predicted protein-encoding genes. One dual-gene and two single-gene deletion mutants of the main CSPs, *Vpa*CspA and *Vpa*CspD, in *V. parahaemolyticus* CHN25 were successfully constructed. Our data demonstrated that *Vpa*CspA was a primary CSP in the bacterium, whereas *Vpa*CspD functioned as a growth inhibitor at 10 °C. Moreover, *VpacspD* gene expression was negatively regulated by *Vpa*CspA. A global-level transcriptomic analysis revealed distinct gene expression profiles among the three mutants. Approximately 12.4% of the expressed genes in *V. parahaemolyticus* CHN25 were significantly altered in the Δ*VpacspA* mutant at 10 °C, including those involved in amino acid degradation, ABC transporters, secretion systems, sulphur metabolism and glycerophospholipid metabolism. The low temperature elicited significant changes in expression of 10.0% of the genes from the Δ*VpacspD* mutant, including genes that were involved in the phosphotransferase system and in nitrogen and amino acid metabolism. The following major altered metabolic pathways in the Δ*VpacspAD* mutant radically differed from those in the single-gene mutants at 10 °C: TCA; PTS; butanoate metabolism; fructose and mannose metabolism; pyruvate, cysteine and methionine metabolism; arginine and proline metabolism; alanine, aspartate and glutamate metabolism; and oxidative phosphorylation. The transcriptome profile comparisons further revealed numerous DEGs that were shared among the three mutants and DERs that were specifically, coordinately and or antagonistically mediated by *Vpa*CspA and *Vpa*CspD at a low temperature. *V. parahaemolyticus* appears to have evolved several molecular strategies with a complex gene regulation network for coping with cold-induced damage. The results from this study improve our understanding of the genetic basis for low-temperature survival of the most common seafood-borne pathogens worldwide.

## Methods

### Bacterial strains, plasmids and culture conditions

*Escherichia coli* DH5α *λpir* (BEINUO Biotech (Shanghai) CO., LD. Shanghai, China) was used as a host strain for DNA cloning. The pDS132 plasmid [50] (a kind gift from Professor Dominique Schneider) was used as a suicide vector to construct the gene deletion mutants. *E. coli* β2155 *λpir* [51] (a kind gift from Professor Weicheng Bei) was used as a donor strain in the conjugation experiments. The pMMB207 plasmid [52] (Biovector Science Lab, Inc., Beijing, China) was used as a expression vector to construct the reverse mutants. *V. parahaemolyticus* CHN25 was isolated and characterised by Song et al. [15], Sun et al. [16] and He et al. [17] and modified for mutant construction by Sun et al. (unpublished). The bacterium was positive for the *tlh* gene but contained no toxic *tdh* and *trh* genes [15]. The *E. coli* strains were routinely incubated in Luria-Bertani (LB) medium (1% NaCl, pH 7.2) [53] at 37 °C, and the *V. parahaemolyticus* strains were grown in LB medium (3% NaCl, pH 8.5). The diaminopimelic acid (DAP) auxotrophic *E. coli* strains were grown in LB medium that contained 0.3 mM DAP (Sigma-Aldrich, MO, USA). The medium was supplemented as needed with chloramphenicol to a final concentration of 30 μg/mL for *E. coli* and 5 μg/mL for *V. parahaemolyticus*. Growth curves were determined as previously described [16].

### Genome sequencing, assembly, gene functional annotation, and comparative genome analysis

Whole-genome sequencing of *V. parahaemolyticus* CHN25 was performed at the Chinese National Human Genome Centre (Shanghai, China) using the Genome Sequencer FLX (GS-FLX) system (Roche, Mannheim, Germany), which yielded 177,497 reads with a genome sequencing depth of 22-fold. The sequencing reads were assembled using the Newbler V2.3 software [54]. Gap closure was performed by primer walking and combinatorial PCR as previously described [55]. The final genome assembly was performed using the Phred-Phrap-Consed software packages [56]. Protein-coding genes were predicted using the EasyGene software [57], and functional assignments were inferred based on standalone Basic Local Alignment Search Tool (BLAST) ( http://www.ncbi.nlm.nih.gov/BLAST) searches against the SWISS-PROT, GenBank, Clusters of Orthologous Groups of proteins (COGs) [58], and Pfam databases [59]. The rRNA genes were annotated using the FgenesB tool (http://softberry.com/), and tRNA genes were detected using the tRNAscan-SE programme [60]. IS elements were identified using the IS Finder [VC41]. Prophage-associated genes were predicted using Prophage finder (http://phast.wishartlab.com/). The clustered regularly interspaced short palindromic repeats (CRISPRs) were identified using the CRISPRFinder [61]. Potential virulence factors were detected using the Virulence Factor database (http://www.mgc.ac.cn/VFs/). Whole genome sequence alignments were performed using MUMmer3.2.3 software (http://www.tigr.org/software/mummer/) [62].

### Deletion of the *VpacspA* and *VpacspD* genes in *V. parahaemolyticus* CHN25

Genomic DNA was prepared using the Biospin Bacteria DNA Extraction Kit (BIOER Technology, Hangzhou, China). Plasmid DNA was isolated using the TaKaRa MiniBEST Plasmid Purification Kit Version 3.0 (Japan TaKaRa BIO, Dalian Company, China). A markerless deletion mutant of the *VpacspA* gene was constructed by homologous recombination (Philippe et al. 2004). Based on the *VpacspA* gene sequence (213 bp, assigned to VpaChn25A_0413) of the *V. parahaemolyticus* CHN25 genome, primer pairs were designed (*cspA*-up-F/*cspA*-up-R and *cspA*-down-F/*cspA*-down-R) to target the upstream (528 bp) and downstream (513 bp) sequences, respectively, of the *VpacspA* gene (see Additional file 4: Table S2). The amplified PCR products were individually digested with corresponding restriction endonucleases (TaKaRa), purified, and ligated into the pDS132 *Xba*I and *Sac*I cloning sites as previously described [50, 63]. The ligated DNA was transformed into *E. coli* DH5α *λpir* competent cells using the heat-shock method [52]. Positive transformants were screened by colony PCR. The recombinant plasmid, pDS132 + Δ*VpacspA,* was subsequently prepared and transformed into DAP auxotroph *E. coli* β2155 competent cells. Plate mating assays were performed using *E. coli* β2155 (pDS132 + Δ*VpacspA*) as the donor and modified *V. parahaemolyticus* CHN25 as the recipient, as previously described [15, 50]. Mating was performed at 37 °C for 12 h on LB plates (1.5% NaCl, pH 7.2) that contained 0.3 mM DAP. Cells that were grown on the mating plates were transferred onto LB plates (3% NaCl, pH 8.5) that contained 5 μg/mL chloramphenicol, which enabled the optimal growth of *V. parahaemolyticus* CHN25. Transconjugants were then inoculated into LB broth (3% NaCl, pH 8.5) without chloramphenicol and incubated overnight; serial dilutions were spread onto the selective LB agar plates, which were supplemented with 10% (wt/vol) sucrose. Exconjugants with successful double crossover deletions of the *VpacspA* gene were screened by colony PCR using the *cspA*-up-exF and *cspA*-down-exR primer pair and confirmed by DNA sequencing. The 219-bp *VpacspD* gene (VpaChn25_1036) deletion was carried out using the method described above with the primer designs listed in Additional file 4: Table S2. Furthermore, the *VpacspD* gene was also deleted from the Δ*VpacspA* mutant to create the dual-gene deleted Δ*VpacspAD* mutant.

### Construction of the reverse mutants of the *VpacspA* and *VpacspD* genes in *V. parahaemolyticus* CHN25

The *VpacspA* gene was amplified from the genomic DNA of *V. parahaemolyticus* CHN25 by PCR with the *cspA*-com-F and -R primers (Additional file 4: Table S2). The PCR product was, digested with corresponding restriction endonucleases (TaKaRa), purified, and ligated into the expression vector pMMB207 at the *Sac*I and *Xba*I cloning sites. The ligated DNA was transformed into *E. coli* DH5α and positive transformants were screened as described above. The recombinant plasmid pMMB207 + *VpacspA* was then prepared and transformed into the Δ*VpacspA* mutant by electrotransformation. The competent cells of the Δ*VpacspA* mutant was prepared according to the method Hamashima et al. [64] with minor modification. Briefly, the Δ*VpacspA* mutant was inoculated into 5 mL Mueller-Hinton Broth (MHB, 3% NaCl, pH7.0) (Beijing Land Bridge Technology Co., Beijing, China) and incubated at 37 °C. The overnight culture was then collected by centrifugation at 2,700 g for 4 min, 4°C, and the cell pellet was suspended and washed with cooled EP buffer (272 mM sucrose, 1 mM MgCl_2_, 7 mM KH_2_PO_4_-Na_2_HPO_4_, pH 7.4) for three times. The washed cells were finally suspended with 8 mL cooled EP buffer, and 200-μL aliquots of the cells were stored at −80 °C. The electrotransformation was performed according to the method [64]. Briefly, 1 μg DNA of the plasmid pMMB207 + *VpacspA* was added into 200 μL competent cells of the Δ*VpacspA* mutant, and incubated on ice for 15 min. The electrotransformation was performed at 25 ms, 1.5 kV,100 Ω,25 μF conditions using the Gene Pluser XCell (Bio-Rad,USA). Subsequently, 500 μL prewarmed MHB (3% NaCl, pH7.0) was quickly added into the electrotransformation mixture, and incubated at 37 °C for 1 h. The cell culture was then spread onto MHB agar plates supplemented with 5 μg/mL chloramphenicol, and cultured at 37 °C overnight. The positive electrotransformant (Δ*VpacspA*-com mutant) were screened by colony PCR with primers *cspA*-com-FR and *tlh*-FR, and confirmed by DNA sequencing analysis. Similarly, the reverse mutant Δ*VpacspD*-com was also constructed with the *cspD*-com-F and -R primers (Table S2) using the same methods described above.

### Illumina RNA sequencing

Bacterial cells were cultured at 10 °C until they reached their logarithmic growth phase and were collected by centrifugation. Total RNA was prepared using the RNeasy Protect Bacteria Mini Kit (QIAGEN Biotech Co. Ltd., Hilden, Germany) and QIAGEN RNeasy Mini Kit (QIAGEN) according to the manufacturer’s protocols. The DNA was removed from the samples with the RNase-Free DNase Set (QIAGEN). Three independently prepared RNA samples were used in each Illumina RNA-sequencing experiment. A wild-type strain that was cultured under identical conditions was used as the control.

The sequencing library construction and Illumina sequencing were conducted at Shanghai Biotechnology Co., Ltd. (Shanghai, China) according to the TruSeq^TM^RNA Sample Preparation Guide (Illumina, San Diego, CA, USA). The abundant 16S and 23S rRNA were depleted using the Ribo-Zero rRNA Removal Kit (Epicentre Biotechnologies, Madison, WI, USA). First-strand cDNA was synthesised using SuperScript II Reverse Transcriptase (Invitrogen, Grand Island, NY, USA) with random hexamer primers. AMPure XP Beads (Beckman Coulter, Beverly, MA, USA) were used to isolate double-stranded cDNA that was synthesised with the Second Strand Master Mix (Invitrogen). The cDNA fragments underwent an end-repair process to convert the overhangs into blunt ends. A single “A” nucleotide was added to the 3’ ends of each blunt fragment to prevent them from ligating to one another during the adapter ligation reaction. The adapters (data not shown) with corresponding single “T” nucleotides on their 3’ ends were ligated, and PCR reactions were performed to enrich the DNA fragments that contained adapter molecules on both ends. Prepared sequencing libraries were quantified with a Qubit^R^ 2.0 Fluorometre (Invitrogen) and validated using the Agilent High-Sensitivity DNA assay on the Agilent Bioanalyser 2100 system (Agilent Technologies, Santa Clara, CA, USA). Clustering of the index-coded samples was performed on a cBot Cluster Generation System using the TruSeq PE Cluster Kit v3-cBot-HS (Illumina). Sequencing was conducted using an Illumina HiSeq 2500 platform, which generated 2 × 100-bp paired-end reads. High quality reads that passed the Illumina quality filters were used for sequence analyses.

### Data analysis

Quality filtration of raw RNA-seq data were performed using the FASTX-Toolkit version 0.0.13 (http://hanonlab.cshl.edu/fastx_toolkit/index.html) to remove the sequencing adapters, identical and low-quality reads, and ribosomal RNA sequences. The resulting clean reads were aligned to the *V. parahaemolyticus* CHN25 genome using the Bowtie2 version 2.0.5 software (http://bowtie-bio.sourceforge.net/bowtie2/index.shtml). Gene transcriptional abundance of assembling transcripts was estimated according to the reads per kilobase of exon model per million mapped reads (RPKM) method described by Mortazavi et al. [65]. The fold-change was determined for each gene by calculating the ratio of the RPKM values between the sample and the control. The genes with criteria fold-changes ≥ 2.0 or ≤ 0.5 and *p*-values < 0.05 relative to the control were defined as DEGs. These DEGs were used for GSEA against the KEGG database (http://www.genome.jp/kegg/), and significantly changed metabolic pathways were identified when the enrichment test p-value fell below 0.05, which was validated by eBioservice (http://sas.ebioservice.com/portal/root/molnet_shbh/index.jsp) (Shanghai Biotechnology Co., Ltd., Shanghai, China) [16].

### Real-time reverse transcription PCR

Selected DEGs and significantly enriched genes in the transcriptome-sequencing analysis were validated by qRT-PCR. Oligonucleotide primers were designed using the Primer 5.0 software (http://www. premierbiosoft.com/) (see Additional file 5: Table S3) and synthesised by Shanghai Sangon Biological Engineering Technology Services Co., Ltd. (Shanghai, China). The conditions that were utilised to grow the cells for the qRT-PCR analysis were identical to those used for Illumina RNA sequencing. The qRT-PCR reactions were performed as previously described [16]. Primer specification was confirmed by agarose gel electrophoresis and melting curve analyses, and qRT-PCR amplification efficiencies (E) were analysed using the Applied Biosystems 7500 software programme (Applied Biosystems, Foster City, CA, USA). The relative expression ratio (*R*) of the target gene was calculated based on E and the crossing point (CP) deviation of the sample versus the control, and it was expressed relative to the reference gene using the delta-delta threshold cycle (C_T_) method as previously described by Pfaffl [66]. The 16S rRNA gene was used as the reference gene, as previously described [67]. All determinants were performed in triplicate.

## Additional files

Additional file 1: Figure S1. Circular maps of the *V. parahaemolyticus* CHN25 chromosomes. (a) and (b) represent the larger and smaller chromosomes of *V. parahaemolyticus* CHN25, respectively. Each circle in the grey lines, except for the two innermost circles, illustrates specific features on the plus (*outer region*) and minus (*inner region*) strands. The lines and boxes in the three outermost circles are coloured according to the COG categories. The circles indicate the following from the outside inwards: first circle, predicted protein-coding genes; second circle, classified essential genes, including cell division, replication, transcription, translation, and amino acid metabolism; third circle, tRNA genes and rRNA operons; fourth circle, GC-skew (*values above zero are red, values below zero are blue*); fifth circle, GC content. (TIF 22070 kb)

Additional file 2: Figure S2. Circular maps of the *V. parahaemolyticus* CHN25 plasmids. (a)-(c): each circle in the grey lines, except for the two innermost circles, illustrates specific features on the plus (*outer region*) and minus (*inner region*) strands. Lines and boxes in the three outermost circles are coloured according to the COG categories. The circles indicate the following from the outside inwards: first circle, predicted protein-coding genes; second circle, GC-skew (*values above zero in red, values below zero in blue*); and third circle, GC content. (TIF 15096 kb)

Additional file 3: Table S1. The DERs in the Δ*VpacspA*, Δ*VpacspD* and Δ*VpacspAD* mutants of *V. parahaemolyticus* CHN25 at low temperatures. (DOC 96 kb)

Additional file 4: Table S2. Oligonucleotide primers for mutant construction used in this study. (DOC 48 kb)

Additional file 5: Table S3. Oligonucleotide primers used for the RT-PCR analysis in this study. (DOC 59 kb)

## Abbreviations

ABC: ATP-binding cassette
BCCTs: Betaine-choline-carnitine transporters
BLAST: basic local alignment search tool
cAMP: 3’-5’-cyclic adenosine monophosphate
COGs: clusters of orthologous groups
CP: crossing point
CRISPRs: clustered regularly interspaced short palindromic repeats
CRP: cAMP receptor protein
CSPs: Cold shock proteins
DAP: Diaminopimelic acid
DEGs: Differentially expressed genes
DHAP: Dihydroxyacetone phosphate
GB: Glycine betaine
G3P: Sn-glycerol-3-phosphate
GSEA: Gene set enrichment analysis
ISs: Insertion sequences
R-M: Restriction and modification
KEGG: Kyoto encyclopaedia of genes and genomes
PTS: Phosphotransferase system
TCA: Tricarboxylic acid cycle
TFP: Type IV pili
TSS: Secretion system types
