# Supplementary figures and images for: Genomic and transcriptomic analyses reveal distinct biological functions for cold shock proteins (VpaCspA and VpaCspD) in Vibrio parahaemolyticus CHN25 during low-temperature survival
Source: BMC Genomics. 2017 Jun 5;18:436. doi: 10.1186/s12864-017-3784-5 (PMC5460551; doi:10.1186/s12864-017-3784-5)

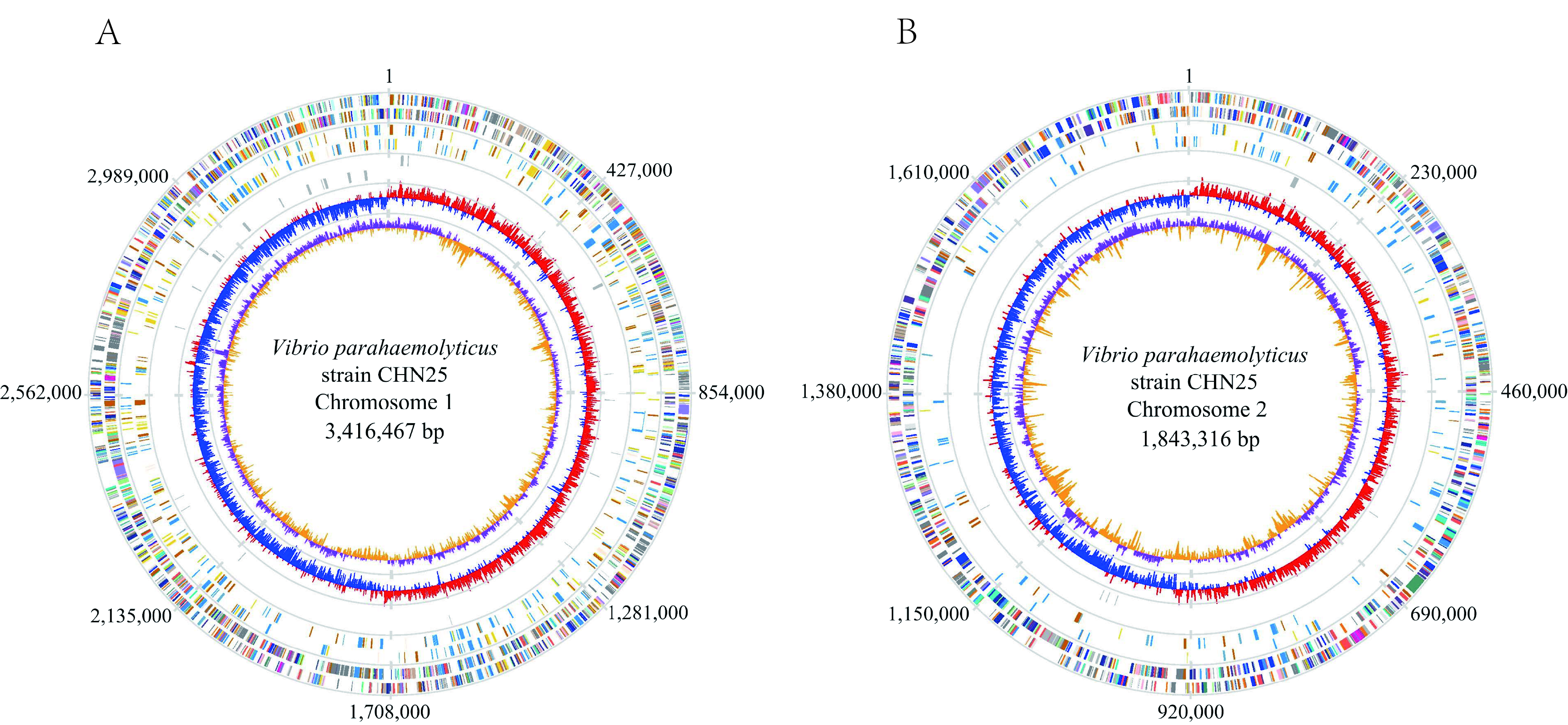

Supplement: Supplementary file 1 — Circular maps of the V. parahaemolyticus CHN25 chromosomes. (a) and (b) represent the larger and smaller chromosomes of V. parahaemolyticus CHN25, respectively. Each circle in the grey lines, except for the two innermost circles, illustrates specific features on the plus (outer region) and minus (inner region) strands. The lines and boxes in the three outermost circles are coloured according to the COG categories. The circles indicate the following from the outside inwards: first circle, predicted protein-coding genes; second circle, classified essential genes, including cell division, replication, transcription, translation, and amino acid metabolism; third circle, tRNA genes and rRNA operons; fourth circle, GC-skew (values above zero are red, values below zero are blue); fifth circle, GC content. (TIF 22070 kb) [file 12864_2017_3784_MOESM1_ESM.tif]

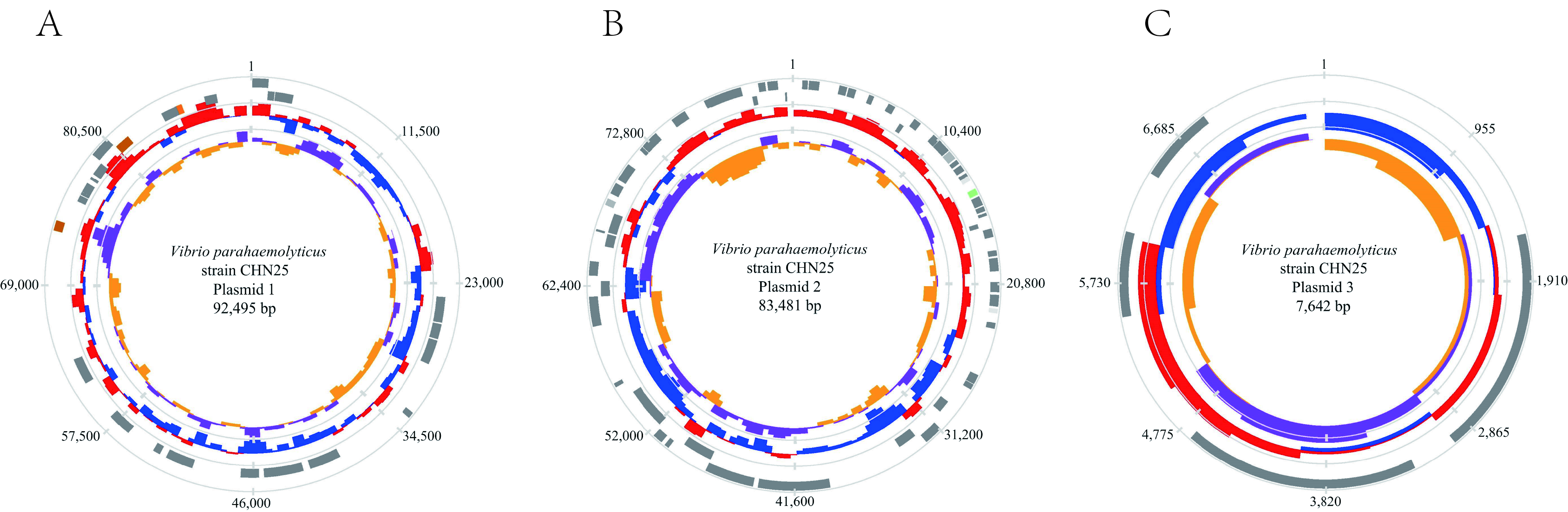

Supplement: Supplementary file 2 — Circular maps of the V. parahaemolyticus CHN25 plasmids. (a)-(c): each circle in the grey lines, except for the two innermost circles, illustrates specific features on the plus (outer region) and minus (inner region) strands. Lines and boxes in the three outermost circles are coloured according to the COG categories. The circles indicate the following from the outside inwards: first circle, predicted protein-coding genes; second circle, GC-skew (values above zero in red, values below zero in blue); and third circle, GC content. (TIF 15096 kb) [file 12864_2017_3784_MOESM2_ESM.tif]
