# Supplementary material for: Genomic and transcriptomic analyses reveal distinct biological functions for cold shock proteins (VpaCspA and VpaCspD) in Vibrio parahaemolyticus CHN25 during low-temperature survival
Source: BMC Genomics. 2017 Jun 5;18:436. doi: 10.1186/s12864-017-3784-5 (PMC5460551; doi:10.1186/s12864-017-3784-5)
Supplement: Supplementary file 3 — The DERs in the ΔVpacspA, ΔVpacspD and ΔVpacspAD mutants of V. parahaemolyticus CHN25 at low temperatures. (DOC 96 kb) [file 12864_2017_3784_MOESM3_ESM.doc]

**Additional file 3: Table S1** The DERs in the Δ*VpacspA*, Δ*VpacspD* and Δ*VpacspAD* mutants of *V. parahemolyticus* CHN25 grown at the low temperature

| **Locus / gene** | **Fold change** | | |  | **Description of encoded protein** |
| --- | --- | --- | --- | --- | --- |
|  | **Δ*VpacspA*** | **Δ*VpacspD*** | **Δ*VpacspAD*** |  |  |
| VpaChn25A_0100 | 0.4597 | - | - | | LysR family transcriptional regulator |
| VpaChn25A_0149 | 0.2213 | 0.3057 | 0.3057 | | Transcriptional regulator CpxR |
| VpaChn25A_0354 | 2.0217 | - | 3.3062 | | LuxR family transcriptional regulator |
| VpaChn25A_0376 | 0.418 | - | - | | Transcriptional regulator |
| VpaChn25A_0470 | 0.3729 | - | - | | Transcriptional regulator |
| VpaChn25A_0559 | 3.0316 | - | 2.5584 | | Transcriptional regulator |
| VpaChn25A_0568 | 0.4784 | - | - | | Transcriptional regulator BetI |
| VpaChn25A_0662 | 2.1305 | - | 2.9477 | | LysR family transcriptional regulator |
| VpaChn25A_0685 | 2.5856 | - | 2.9419 | | Transcriptional regulator |
| VpaChn25A_0878 | 2.1486 | - | - | | AraC family transcriptional regulator |
| VpaChn25A_0888 | 0.4123 | - | - | | ArsR family transcriptional regulator |
| VpaChn25A_0975 | 2.1798 | - | - | | Two-component response regulator |
| VpaChn25A_1014 | 2.1015 | - | - | | LysR family transcriptional regulator |
| VpaChn25A_1035 | 2.8253 | - | - | | Transcriptional regulator |
| VpaChn25A_1109 | 8.5353 | - | - | | Transcriptional regulator |
| VpaChn25A_1311 | 2.4363 | 7.5691 | 4.1119 | | Transcriptional regulator |
| VpaChn25_0017 | 0.3406 | - | - | | LysR family transcriptional regulator |
| VpaChn25_0025 | 0.3492 | - | - | | DNA-binding transcriptional regulator IlvY |
| VpaChn25_0582 | 2.0347 | 2.0443 | - | | LysR family transcriptional regulator |
| VpaChn25_1130 | 2.3094 | 2.7924 | 3.5489 | | Transcriptional regulator |
| VpaChn25_1250 | 2.619 | 3.0609 | - | | Response regulator |
| VpaChn25_1640 | 4.2358 | 2.2896 | - | | GntR family transcriptional regulator |
| VpaChn25_1667 | 0.2974 | - | - | | Transcriptional regulator |
| VpaChn25_2042 | 3.8076 | 8.2123 | - | | Regulatory protein |
| VpaChn25_2454 | 0.3122 | - | - | | Transcriptional regulator PdhR |
| VpaChn25_2483 | 0.2654 | - | - | | Recombination regulator RecX |
| VpaChn25_2533 | 0.3545 | - | - | | Iron-regulated virulence regulatory protein |
| VpaChn25_2717 | 2.3774 | 2.9171 | - | | cAMP-regulatory protein |
| VpaChn25_2920 | 0.403 | - | - | | LysR family transcriptional regulator |
| VpaChn25_PB39 | 0.4779 | - | - | | Phage transcriptional regulator AlpA |
| VpaChn25A_0686 | - | 0.4764 | - | | Fimbrial protein Z transcriptional regulator |
| VpaChn25A_0772 | - | 0.4825 | 0.4968 | | Regulatory protein UhpC |
| VpaChn25A_1000 | - | 0.4499 | - | | Two-component response regulator |
| VpaChn25A_1310 | - | 4.2109 | 2.6367 | | PTS system nitrogen regulatory subunit IIA |
| VpaChn25A_1361 | - | 2.891 | - | | MerR family transcriptional regulator |
| VpaChn25A_1453 | - | 0.403 | - | | Transcriptional regulator |
| VpaChn25_0344 | - | 0.2185 | 0.2147 | | DeoR family transcriptional regulator |
| VpaChn25_0707 | - | 0.3832 | - | | Putative transcriptional regulator, Nlp |
| VpaChn25_0718 | - | 0.454 | 0.4503 | | Positive regulator of late transcription |
| VpaChn25_1251 | - | 2.8939 | - | | Response regulator |
| VpaChn25_1651 | - | 0.4166 | 0.4266 | | Type III secretion regulator |
| VpaChn25_1688 | - | 0.4034 | - | | Type III secretion regulator ExsD |
| VpaChn25_1689 | - | 0.2431 | 0.4823 | | Type III secretion regulator ExsA |
| VpaChn25_1692 | - | 0.3474 | 0.3079 | | Type III secretion regulator ExsE |
| VpaChn25_2510 | - | 0.4588 | - | | Sigma-E factor negative regulatory protein RseA |
| VpaChn25A_0113 | - | - | 2.529 | | Transcriptional regulator |
| VpaChn25A_0177 | - | - | 2.2751 | | LysR family transcriptional regulator |
| VpaChn25A_0351 | - | - | 2.2776 | | Transcriptional regulator |
| VpaChn25A_0644 | - | - | 2.0942 | | Arylsulfatase regulator |
| VpaChn25A_0655 | - | - | 2.2222 | | Sensor histidine kinase/response regulator LuxN |
| VpaChn25A_0682 | - | - | 2.5524 | | Two-component response regulator |
| VpaChn25A_0683 | - | - | 2.0246 | | Fimbrial protein Z transcriptional regulator |
| VpaChn25A_1283 | - | - | 2.45 | | AraC family transcriptional regulator |
| VpaChn25A_1452 | - | - | 2.1053 | | Transcriptional regulator |
| VpaChn25A_1464 | - | - | 2.5214 | | DNA-binding transcriptional regulator YidZ |
| VpaChn25A_1598 | - | - | 2.2637 | | Transcriptional regulator |
| VpaChn25A_1612 | - | - | 2.0069 | | AraC family transcriptional regulator |
| VpaChn25A_1614 | - | - | 2.1684 | | LuxR family transcriptional regulator |
| VpaChn25_0239 | - | - | 0.3216 | | Ribonuclease activity regulator protein RraA |
| VpaChn25_0871 | - | - | 2.0144 | | LysR family transcriptional regulator |
| VpaChn25_1292 | - | - | 2.3705 | | Transcriptional regulator |
| VpaChn25_1330 | - | - | 0.3362 | | Transcription regulator |
| VpaChn25_1727 | - | - | 2.0095 | | LysR family transcriptional regulator |
| VpaChn25_2165 | - | - | 0.4253 | | Nitrogen regulatory protein P-II |
| VpaChn25_2480 | - | - | 0.4277 | | Carbon storage regulator |

“-”, not significantly changed.
