# Supplementary material for: Genomic and transcriptomic analyses reveal distinct biological functions for cold shock proteins (VpaCspA and VpaCspD) in Vibrio parahaemolyticus CHN25 during low-temperature survival
Source: BMC Genomics. 2017 Jun 5;18:436. doi: 10.1186/s12864-017-3784-5 (PMC5460551; doi:10.1186/s12864-017-3784-5)
Supplement: Supplementary file 4 — Oligonucleotide primers for mutant construction used in this study. (DOC 48 kb) [file 12864_2017_3784_MOESM4_ESM.doc]

**Additional file 4: Table S2** Oligonucleotide primers for mutant construction used in this study

| **Primer** | **Sequence (5’-3’)^a^** | **Gene location** | **PCR product (bp)** ^b^ |
| --- | --- | --- | --- |
| *cspA*-up-F | GCTCTAGACGTTCGTTGTTTTACAGCGGCAGCATTT | VpaChn25C2_446210-446791 | 599 |
| *cspA*-up-R | AGCGGCCGCGACTGCTTTTCTTGGTGCAGCATGT |  |  |
| *cspA*-down-F | GGCGGCCGCAAAAACCTCTGAATTAAAAACAT | VpaChn25C2_447005-447517 | 529 |
| *cspA*-down-R | CGAGCTCTCAGCGGTAGGTTGCGATGGAAT |  |  |
| *cspA*-F | TTATAGAGGAGTAACTTCAGTCG | VpaChn25C2_447004-446792 | 213 |
| *cspA*-R | ATGTCTGCTAAAGTAACTGG |  |  |
| *cspA*-up-exF | GCGGAGTGGAAAGGTAGGTGG | VpaChn25C2_446086-447761 | 1463 (1676) |
| *cspA*-down-exR | GCGACCTGCCGCCATCCATT |  |  |
| *cspD*-up-F | GCTCTAGAGCATTTAAGCTCAGTAACTGGCAATGC | VpaChn25C1_1175470-1175975 | 523 |
| *cspD*-up-R | AGCGGCCGCATTGAGTGCTGTCGTAGAAACCATT |  |  |
| *cspD*-down-F | GGCGGCCGCGCTTATACATCCCTCATGCATTT | VpaChn25C1_1176195-1176739 | 561 |
| *cspD*-down-R | CGAGCTCGCGGGCGCGCAAATAAAAGATGG |  |  |
| *cspD*-F | TTACTTGGCTTGGGCTTCA | VpaChn25C1_1176194-1175976 | 219 |
| *cspD*-R | ATGGCTACAGGTACAGTAAAGTGG |  |  |
| *cspD*-up-exF | ACTATTTCTGTAACGGGTAACG | VpaChn25C1_1175237-1176936 | 1481 (1700) |
| *cspD*-down-exR | GAAGATCAGAGTCCGGAGTG |  |  |
| *cspA*-com-F | CGAGCTCTTATAGAGGAGTAACTTCAGTCG | VpaChn25C2_447004-446792 | 228 |
| *cspA*-com-R | GCTCTAGAATGTCTGCTAAAGTAACTGG |  |  |
| *cspD*-com-F | CGAGCTCTTACTTGGCTTGGGCTTCA | VpaChn25C1-1175976-1176215 | 255 |
| *cspD*-com-R | GCTCTAGAATGCATGAGGGATGTATAAGC |  |  |
| *tlh-*F | AAAGCGGATTATGCAGAAGCACTG | VpaChn25C2_245282-245729 | 448 |
| *tlh-*R | ACTTTCTAGCATTTTCTCTGC |  |  |

^a^ underlined sequences represent the recognition sites of restriction endonucleases *Xba*I and *Not*I, *Not*I and *Sac*I that were introduced via the forward and reverse primers, respectively.

^b^ data in the brackets represent the product containing targeted genes.
