# Supplementary material for: Genomic and transcriptomic analyses reveal distinct biological functions for cold shock proteins (VpaCspA and VpaCspD) in Vibrio parahaemolyticus CHN25 during low-temperature survival
Source: BMC Genomics. 2017 Jun 5;18:436. doi: 10.1186/s12864-017-3784-5 (PMC5460551; doi:10.1186/s12864-017-3784-5)
Supplement: Supplementary file 5 — Oligonucleotide primers used for the RT-PCR analysis in this study. (DOC 59 kb) [file 12864_2017_3784_MOESM5_ESM.doc]

**Additional file 5: Table S3** Oligonucleotide primers used for the RT-PCR analysis in this study

| **Locus / gene in**  ***V. parahemolyticus* CHN25** | **Sequence (5′ to 3′)** | **PCR product length**  **(bp)** |
| --- | --- | --- |
| VpaChn25A_0149-F | AACCATTTGTAATACCGCCC | 266 |
| VpaChn25A_0149-R | AACTGTAGCCTTTGCCTCGA |
| VpaChn25A_0188-F | TGGTGTTCCAGTTGGGGCGGT | 293 |
| VpaChn25A_0188-R | CTTTGGTTGCTTTGTGGATGCCTTG |
| VpaChn25A_0303-F | AGAATACGGTCGGTTTGACTCCCT | 232 |
| VpaChn25A_0303-R | GAAGTCTTTGAAGAGCTAGCGC |
| VpaChn25A_0561-F | CATCTCCGACTCAAAACAATGGCTA | 224 |
| VpaChn25A_0561-R | AAAGCAGTATCGCCAACTCATCGTG |
| VpaChn25A_0568-F | CTTCGTGCGTTGCCAGCAGATG | 286 |
| VpaChn25A_0568-R | TGAGCGTGCCGCGTAGCCATAT |
| VpaChn25A_1312-F | CCTAATGGACGGTCTGACTGC | 279 |
| VpaChn25A_1312-R | GCTTTACCTGCTTCGGTTTCT |
| VpaChn25A_1313-F | TGGAAGGCGAACAGAAAGAGATGG | 351 |
| VpaChn25A_1313-R | CGATTGGTGCAAGCAGTAGGGTA |
| VpaChn25A_1398-F | ACCCACAAGGTCGAAAGAAAGCG | 241 |
| VpaChn25A_1398-R | CGTATCTGGCGAAGGTCACATCA |
| VpaChn25A_1399-F | TACAGGGAATGAGAAGCCGAT | 243 |
| VpaChn25A_1399-R | CCAGCAATCGTAAACGCTTCTAT |
| VpaChn25_0112-F | GCGTTTAGGGTCGGTAGAGAGT | 252 |
| VpaChn25_0112-R | TGGGAAGAGCAAGGTCTGAAC |
| VpaChn25_0068-F | CTATGCCAGCAAACAGCCTTACAGA | 300 |
| VpaChn25_0068-R | CTTGCCAGTTCGGTGTGTAGTTATC |
| VpaChn25_0669-F | ACCTTGGTGTGGATGCGAT | 253 |
| VpaChn25_0669-R | TAGTAGTCACGGTATGGGCTGT |
| VpaChn25_1640-F | GTTCGGAGGTGGCGTAGTTT | 264 |
| VpaChn25_1640-R | TGTCCAGTCAAGGTTTCAGC |
| VpaChn25_1642-F | GAGAGTTTTATCGCGACCTA | 286 |
| VpaChn25_1642-R | TTATCGGACATCACCTTGCC |
| VpaChn25_1716-F | CCGCTCCATGCAGACAAATACA | 256 |
| VpaChn25_1716-R | CGTCCAACTGATGCTCAACCAC |
| VpaChn25_2248-F | GGGAAATCAGTATTGTATGGGGC | 306 |
| VpaChn25_2248-R | AAAGAGAAGCGTGTGGGTAAG |
| VpaChn25_2249-F | AGCGAAAAACACATACGGCACAG | 364 |
| VpaChn25_2249-R | CAGAGTCGCTCGGATAATGTGGT |
| VpaChn25_2988-F | TCTTTGTCGTATTGGCGTAGTAGC | 312 |
| VpaChn25_2988-R | CTTTGAGGTGGATATGTGGGATG |
| 16S rRNA -F | GAAGAAGCACCGGCTAACTCC | 101 |
| 16S rRNA -R | AACAAACCACCTGCATGCG |
